# Supplementary material for: Long-term health outcomes of people with reduced kidney function in the UK: A modelling study using population health data
Source: PLoS Med. 2020 Dec 16;17(12):e1003478. doi: 10.1371/journal.pmed.1003478 (PMC7769604; doi:10.1371/journal.pmed.1003478)
Supplement: S1 Text — (DOCX) [file pmed.1003478.s002.docx]

**fS1 Text: Supplementary tables, figures and methods**

**Long-term health outcomes of people with reduced kidney function: a policy model derived from a large UK population cohort**

**Table of Content**

[**Table A. Health-related quality of life related to reduced kidney function and cardiovascular events based on the United Kingdom EQ-5D-3L value set** 2](#_Toc57216191)

[**Table B. Annual healthcare costs in patients with reduced kidney function in the United Kingdom** 4](#_Toc57216192)

[**Fig A. Open cohort of patients with at least two measurements of eGFR<90 mL/min/1.73m^2^ at least 90 days apart derived from the Clinical Practice Research Datalink (CPRD)** 5](#_Toc57216193)

[**Table C. Baseline characteristics of the estimation cohort of patients with reduced kidney function in CPRD, by eGFR category at study entry** 6](#_Toc57216194)

[**Table D. Baseline characteristics of the validation cohort of patients with reduced kidney function in CPRD, by eGFR at study entry** 8](#_Toc57216195)

[**Table E. Study participants (%) experiencing adverse events of interest during follow-up, by eGFR at cohort entry** 10](#_Toc57216196)

[**Table F. Risk equations for nonvascular death among patients with reduced kidney function, CPRD estimation cohort** 11](#_Toc57216197)

[**Table G. Risk equations for heart failure hospital admission among patients with reduced kidney function, CPRD estimation cohort** 12](#_Toc57216198)

[**Fig B. Comparison of cumulative risks predicted by the model and observed Kaplan-Meier risks in the validation cohort, for nonvascular death and first heart failure hospitalisation and by eGFR category at cohort entry** 14](#_Toc57216199)

[**Fig C. Comparison of cumulative risks predicted by the model and observed Kaplan-Meier risks in the validation cohort, for the combined endpoint of vascular death, stroke or myocardial infarction, by eGFR category at cohort entry and practice location** 15](#_Toc57216200)

[**Table H. Harrel’s c-index (95% confidence intervals) assessing the discrimination of the CKD-CVD policy model at 5 years, by patient’s eGFR stage at cohort entry** 18](#_Toc57216201)

[**Table I. Predicted life expectancy and quality-adjusted life years without cardiovascular prevention treatments, by cardiovascular disease history, age and eGFR category at cohort entry** 19](#_Toc57216202)

[**Table J. Predicted life years and quality-adjusted life years gained (a) with 2013 cardiovascular prevention medications use compared to no use, and (b) additional gains with optimal medication use, by cardiovascular disease history, age and eGFR category at cohort entry** 21](#_Toc57216203)

[**Methods A.** 26](#_Toc57216204)

## **Table A. Health-related quality of life related to reduced kidney function and cardiovascular events based on the United Kingdom EQ-5D-3L value set**

| **Covariate** | **Effect on quality of life, as measured by EQ-5D-3L utility (standard error)** | **Source** |
| --- | --- | --- |
| Intercept^*^ | 0.838 (0.009) | Regression model using Health Survey for England (HSE) data^1^ |
| **Adjustment for patient‘s characteristic at cohort entry** | | |
| Male gender | 0.030 (0.003) | Regression model using HSE data^1^ |
| Current smoker | -0.056 (0.003) | Regression model using HSE data^1^ |
| With diabetes (type I or type II) | -0.064 (0.007) | Regression model using HSE data^1^ |
| BMI, kg/m^2^ (ref: <25) |  |  |
| ≥25, ≤30 | 0.007 (0.003) | Regression model using HSE data^1^ |
| >30 | -0.055 (0.004) | Regression model using HSE data^1^ |
| History of cardiovascular disease | -0.070 (0.010) | Schlackow et al. (2017)^2^ |
| **Adjustment for patient’s characteristics during annual periods** | | |
| Age (per 10 years) | -0.027 (0.001) | Regression model using HSE data^1^ |
| Renal function decrement  (ref: eGFR 60-89 ml/min/1.73m^2^ [G2]) | | |
| eGFR 45-59 ml/min/1.73m^2^ (G3a) | -0.004 (0.009) | Park et al. (2016)^3^ |
| eGFR 30-44 ml/min/1.73m^2^ (G3b) | -0.036 (0.011) | Park et al. (2016)^3^ |
| eGFR ≤29 ml/min/1.73m^2^ (G4/5), not on RRT | -0.058 (0.019) | Park et al. (2016)^3^ |
| On dialysis | -0.111 (0.021) | Schlackow et al. (2017)^2^ |
| With renal transplant | -0.004 (0.009) | Schlackow et al. (2017)^2^ |
| Previous cardiovascular events within study^+^  (ref: none) | | |
| MI in current year, no previous stroke | 0.760 (0.018) | NICE CG181 Lipid modification  Cardiovascular risk assessment and the modification of  blood lipids for the primary and secondary prevention  of cardiovascular disease (2014)^4^ |
| MI in a previous year, no previous stroke | 0.880 (0.018) |  |
| Stroke in the current year | 0.628 (0.040) |  |
| Stroke in a previous year | 0.628 (0.040) |  |
| Heart failure hospitalisation in current or a previous year | 0.682 (0.020) |  |

BMI, body-mass index, clinical guidelines; CG eGFR, estimated glomerular filtration rate; HSE, health survey for England; MI, myocardial infarction; NICE, National Institute for Health and Care Excellence; RRT, renal replacement therapy

^*^The intercept term corresponds to the quality of life (ie, EQ-5D utility) of a 64-year-old female, non-smoker, with a BMI of <25 kg/m^2^, without diabetes or prior cardiovascular disease and eGFR 60-89 ml/min/1.73 m^2^

^+^multipliers to utility in absence of cardiovascular event in years with particular cardiovascular event history

^1^Quality of life for individuals without cardiovascular and kidney diseases was derived using Health Survey for England data available at <https://digital.nhs.uk/data-and-information/publications/statistical/health-survey-for-england>. Data on participants with no previous cardiovascular disease from years 2003, 2006 and 2011 were used to relate participants’ age, gender, smoking status, diabetes and BMI to their quality of life utility measured using UK EQ-5D-3L tariff (Dolan P. Modeling valuations for EuroQol health states. Med Care. 1997;35(11):1095-108.). ^2^Schlackow I, Kent S, Herrington W et al, on behalf of the SHARP Collaborative Group.A policy model of cardiovascular disease in moderate-to-advanced chronic kidney disease. Heart 2017;103:1880-1890 ^3^Park JI, Baek H and Jung HH. CKD and Health-Related Quality of Life: The Korea National Health and Nutrition Examination Survey American Journal of Kidney Disease 2016 67(6):851-860 ^4^ National Clinical Guideline Centre (UK). Lipid Modification: Cardiovascular Risk Assessment and the Modification of Blood Lipids for the Primary and Secondary Prevention of Cardiovascular Disease. NICE Clinical Guidelines, No. 181. London: National Institute for Health and Care Excellence (UK); 2014– Appendix L, page 587, Table 81

## **Table B. Annual healthcare costs in patients with reduced kidney function in the United Kingdom**

|  | **Costs (£2017)**  **(95% CI)** | **Source** |
| --- | --- | --- |
| **Annual hospital case costs in the absence of cardiovascular complications** | | |
| eGFR 60-89 ml/min/1.73m^2^ (G2)^a^ | 425 (364, 487) | Kent et al. (2015)^1^ |
| eGFR 45-59 ml/min/1.73m^2^ (G3a)^a^ | 425 (364, 487) | Kent et al. (2015)^1^ |
| eGFR 30-44 ml/min/1.73m^2^ (G3b) | 425 (364, 487) | Kent et al. (2015)^1^ |
| eGFR 15-29 ml/min/1.73m^2^ (G4) | 415 (362, 468) | Kent et al. (2015)^1^ |
| eGFR <15 ml/min/1.73m^2^ (G5) not on RRT | 554 (474, 635) | Kent et al. (2015)^1^ |
| On maintenance dialysis^b^ | 24,608 (24,508, 24,709) | Kent et al. (2015)^1^ |
| With renal transplant^c^ | 1,211 (1,032, 1,390) | Kent et al. (2015)^1^ |
| With diabetes (Type I or Type II) | 180 (57, 304) | Kent et al. (2015)^1^ |
| **Additional annual hospital care costs associated with death or cardiovascular**  **complications** | | |
| Nonvascular death in the current year | 1,467 (1,076, 1,860) | Kent et al. (2015)^1^ |
| Vascular death in the current year | 1,200 (495, 1,903) | Kent et al. (2015)^1^ |
| Nonfatal stroke in the current year | 3,286 (2,821, 3,751) | Kent et al. (2016)^2^ |
| Nonfatal MI in the current year | 3,544 (3,059, 4,030) | Kent et al. (2016)^2^ |
| Nonfatal stroke or MI in an earlier year | 196 (124, 269) | Kent et al. (2016)^2^ |
| Heart failure hospitalisation in the current year | 3,059 (2,635, 3,482) | Kent et al. (2016)^2^ |
| Cardiovascular disease at cohort entry | 181 (60, 302) | Kent et al. (2016)^2^ |

CI, confidence interval; eGFR, estimated glomerular filtration rate; MI, myocardial infarction; RRT, renal replacement therapy

^a^In lieu of other evidence, the annual healthcare costs in G2 and G3a were assumed same as costs in G3b.

^b^Cost of dialysis initiated in an earlier year was assumed

^c^Cost of transplant from an earlier year was assumed

^1^Kent S, Schlackow I, Lozano-Kuehne et al, on behalf of the SHARP Collaborative Group. What is the impact of chronic kidney disease stage and cardiovascular disease on the annual cost of hospital care in moderate-to-severe kidney disease? BMC Nephrology (2015) 14:65

^2^Kent S, Haynes R, Hopewell JC et al, on behalf of HPS2-THRIVE Collaborative Group. Effects of Vascular and Nonvascular Adverse Events and of Extended-Release Niacin with Laropiprant on Health and Healthcare Costs. Circ Cardiovasc Qual Outcomes (2016) 9:348-354

## **Fig A. Open cohort of patients with at least two measurements of eGFR<90 mL/min/1.73m^2^ at least 90 days apart derived from CPRD**

Footnote: CPRD, Clinical Practice Research Datalink; eGFR, estimated glomerular filtration rate

## **Table C. Baseline characteristics of the estimation cohort of patients with reduced kidney function in CPRD, by eGFR category at study entry**

|  | **eGFR 60-89 ml/min/1.73m^2^**  **(G2)** | **eGFR 45-59**  **ml/min/1.73m^2^**  **(G3a)** | **eGFR 30-44**  **ml/min/1.73m^2^**  **(G3b)** | **eGFR 15-29**  **ml/min/1.73m^2^**  **(G4)** | **eGFR <15**  **ml/min/1.73m^2^**  **(G5) not on RRT** |
| --- | --- | --- | --- | --- | --- |
| N | 592,179 | 142,051 | 46,724 | 10,495 | 1,816 |
| Age, years | 60 (13) | 73 (11) | 80 (10) | 81 (12) | 71 (16) |
| Male | 279,153 (47%) | 54,090 (38%) | 16,110 (34%) | 3,954 (38%) | 1,005 (55%) |
| Smoking status |  |  |  |  |  |
| Current smoker | 108,819 (18%) | 17,993 (13%) | 5,062 (11%) | 1,164 (11%) | 265 (15%) |
| Previous smoker | 161,151 (27%) | 43,406 (31%) | 14,660 (31%) | 3,254 (31%) | 575 (21%) |
| BMI, kg/m^2^ | 28 (6) | 27 (6) | 27 (6) | 27 (6) | 27 (8) |
| Systolic blood pressure, mmHg | 135 (18) | 140 (18) | 140 (20) | 138 (21) | 138 (23) |
| Diastolic blood pressure, mmHg | 80 (10) | 78 (10) | 76 (11) | 75 (12) | 76 (13) |
| HDL cholesterol (mmol/L) | 1.4 (0.4) | 1.5 (0.4) | 1.4 (0.4) | 1.4 (0.5) | 1.3 (0.5) |
| LDL cholesterol (mmol/L) | 3.2 (1.0) | 3.1 (1.1) | 2.9 (1.1) | 2.8 (1.1) | 2.6 (1.2) |
| Total cholesterol (mmol/L) | 5.3 (1.1) | 5.2 (1.2) | 5.0 (1.2) | 4.9 (1.3) | 4.6 (1.3) |
| Cardiovascular disease history |  |  |  |  |  |
| Coronary disease | 60,890 (10%) | 27,925 (20%) | 14,258 (31%) | 4,098 (39%) | 705 (39%) |
| Cerebrovascular disease | 39,577 (7%) | 16,099 (11%) | 8,487 (18%) | 2,557 (24%) | 541 (30%) |
| Heart failure | 8,153 (1%) | 6,014 (4%) | 5,048 (11%) | 2,026 (19%) | 327 (18%) |
| Family history of coronary heart disease | 108,364 (18%) | 21,305 (15%) | 5,436 (12%) | 1,044 (10%) | 172 (9%) |
| Diabetes |  |  |  |  |  |
| Type I | 3,073 (1%) | 336 (0%) | 177 (0%) | 74 (1%) | 57 (3%) |
| Type II | 58,816 (10%) | 17,914(13%) | 7,887(17%) | 2,283 (22%) | 395 (22%) |
| Albuminuria status |  |  |  |  |  |
| Not measured | 340,046 (57%) | 76,633 (54%) | 24,418(52%) | 5,682(54%) | 1,159(64%) |
| Normoalbuminuria | 242,639 (41%) | 61,340 (43%) | 19,682 (42%) | 3,692 (35%) | 401 (22%) |
| Microalbuminuria | 7,482 (1%) | 3,133 (2%) | 1,843(4%) | 659 (6%) | 73 (4%) |
| Macroalbuminuria | 2,012 (0%) | 945 (1%) | 781 (2%) | 462 (4%) | 183 (10%) |
| Medication intake |  |  |  |  |  |
| On statins^1^ | 132,082 (22%) | 44,038 (31%) | 15,977(34%) | 3,683 (35%) | 676 (37%) |
| On antihypertensives^1^ | 139,666 (24%) | 51,050 (36%) | 22,485 (48%) | 5,573 (53%) | 868 (48%) |
| On antiplatelets / anticoagulants^1^ | 108,364 (17%) | 21,305 (34%) | 5,436 (46%) | 1,044 (49%) | 172 (42%) |
| Townsend multiple socioeconomic deprivation quintile | | | | | |
| First (least deprived) | 139,596 (24%) | 31,851 (22%) | 9,460 (20%) | 2,000 (19%) | 322 (18%) |
| Second | 139,207 (24%) | 34,193 (24%) | 1,0879 (23%) | 2,408 (23%) | 347 (19%) |
| Third | 123,443 (21%) | 30,143 (21%) | 10,115 (22%) | 2,335 (22%) | 416 (23%) |
| Fourth | 110,675 (19%) | 26,643 (19%) | 9,207 (20%) | 2,080 (20%) | 388 (21%) |
| Fifth (most deprived) | 77,677 (13%) | 18,907 (13%) | 6,989 (15%) | 1,658 (16%) | 340 (19%) |
| Region |  |  |  |  |  |
| East Midlands | 22,534 (4%) | 5,775 (4%) | 1,861 (4%) | 415 (4%) | 63 (3%) |
| East of England | 70,647 (12%) | 20,431 (14%) | 6,357 (14%) | 1,308 (12%) | 234 (13%) |
| London | 83,576 (14%) | 18,532 (13%) | 5,515 (12%) | 1,157 (11%) | 279 (15%) |
| North East | 15,245 (3%) | 3,665 (3%) | 1,216 (3%) | 241 (2%) | 42 (2%) |
| North West | 70,413 (12%) | 18,391 (13%) | 5,993 (13%) | 1,312 (13%) | 214 (12%) |
| South Central | 72,082 (12%) | 15,953 (11%) | 5,356 (11%) | 1,359 (13%) | 208 (11%) |
| South East Coast | 94,097 (16%) | 20,988 (15%) | 6,865 (15%) | 1,524 (15%) | 256 (14%) |
| South West | 77,791 (13%) | 17,994 (13%) | 6,107 (13%) | 1,453 (14%) | 257 (14%) |
| West Midlands | 57,352 (10%) | 12,167 (9%) | 4,692 (10%) | 1,094 (10%) | 178 (10%) |
| Yorkshire & The Humber | 28,442 (5%) | 8,155 (6%) | 2,762 (6%) | 632 (6%) | 85 (5%) |

BMI, body mass index; CKD, chronic kidney disease; CPRD, Clinical Practice Research Datalink; eGFR, estimated glomerular filtration rate; HDL, high-density lipoprotein; LDL, low-density lipoprotein; RRT, renal replacement therapy; SD, standard deviation.

Values presented are means (SD) or number (percentage).

^1^ Defined as having had at least 2 prescriptions by the cohort entry date, with at least one prescription ≤84 days before the cohort entry

## **Table D. Baseline characteristics of the validation cohort of patients with reduced kidney function in CPRD, by eGFR at study entry**

|  | **eGFR 60-89 ml/min/1.73m^2^**  **(G2)** | **eGFR 45-59**  **ml/min/1.73m^2^**  **(G3a)** | **eGFR 30-44**  **ml/min/1.73m^2^**  **(G3b)** | **eGFR 15-29**  **ml/min/1.73m^2^**  **(G4)** | **eGFR <15**  **ml/min/1.73m^2^**  **(G5) not on RRT** |
| --- | --- | --- | --- | --- | --- |
| N | 225,468 | 64,007 | 21,368 | 4,730 | 710 |
| Age, years | 60 (13) | 73 (11) | 80 (10) | 81 (11) | 72 (14) |
| Male | 121,036 (47%) | 24,488 (38%) | 7,148 (33%) | 1,824 (29%) | 371 (52%) |
| Smoking status |  |  |  |  |  |
| Current smoker | 46,065 (18%) | 7,968 (12%) | 2,302 (11%) | 501 (11%) | 108 (15%) |
| Previous smoker | 71,122 (28%) | 20,052 (31%) | 6,814 (32%) | 1,521 (32%) | 235 (33%) |
| BMI, kg/m^2^ | 28 (6) | 27 (5) | 27 (6) | 27 (5) | 27 (6) |
| Systolic blood pressure, mmHg | 136 (18) | 140 (18) | 140 (20) | 139 (22) | 139 (22) |
| Diastolic blood pressure, mmHg | 80 (10) | 79 (10) | 77 (11) | 75 (12) | 77 (13) |
| HDL cholesterol (mmol/L) | 1.5 (0.4) | 1.5 (0.4) | 1.5 (0.5) | 1.4 (0.4) | 1.3 (0.4) |
| LDL cholesterol (mmol/L) | 3.2 (1.0) | 3.1 (1.1) | 2.9 (1.1) | 2.8 (1.1) | 2.7 (1.3) |
| Total cholesterol (mmol/L) | 5.3 (1.1) | 5.2 (1.2) | 5.1 (1.2) | 4.9 (1.3) | 4.7 (1.4) |
| Cardiovascular disease history |  |  |  |  |  |
| Coronary disease | 26,384 (10%) | 12,572 (20%) | 6,550 (31%) | 1,899 (40%) | 265 (37%) |
| Cerebrovascular disease | 17,332 (7%) | 7,192 (11%) | 3,859 (18%) | 1,179 (25%) | 234 (33%) |
| Heart failure | 3,552 (1%) | 2,690 (4%) | 2,238 (10%) | 918 (19%) | 141 (20%) |
| Family history of coronary heart disease | 47,471 (19%) | 10,265 (16%) | 2,598 (12%) | 500 (11%) | 70 (10%) |
| Diabetes |  |  |  |  |  |
| Type I | 1,310 (1%) | 176 (0%) | 52 (0%) | 32 (1%) | 21 (3%) |
| Type II | 24,636 (10%) | 7,909 (12%) | 3,350 (17%) | 944 (20%) | 159 (22%) |
| Albuminuria status |  |  |  |  |  |
| Not measured | 143,618 (56%) | 33,364 (52%) | 10,950 (51%) | 2,596 (55%) | 463 (65%) |
| Normoalbuminuria | 107,334 (42%) | 28,692 (45%) | 9,188 (43%) | 1,663 (35%) | 139 (20%) |
| Microalbuminuria | 3,517 (1%) | 1,512 (2%) | 898 (4%) | 278 (6%) | 31 (4%) |
| Macroalbuminuria | 999 (0%) | 439 (1%) | 332 (2%) | 193 (4%) | 77 (11%) |
| Medication intake |  |  |  |  |  |
| On statins^1^ | 57,349 (22%) | 20,114 (31%) | 7,336 (34%) | 1,662 (35%) | 302 (43%) |
| On antihypertensives^1^ | 60,633 (24%) | 22,924 (36%) | 10,087 (47%) | 2,488 (53%) | 339 (48%) |
| On antiplatelets / anticoagulants^1^ | 44,399 (17%) | 21,908 (34%) | 9,952 (47%) | 2,437 (52%) | 315 (45%) |
| Townsend multiple socioeconomic deprivation quintiles | | | | | |
| First (least deprived) | 65,467 (26%) | 15,824 (25%) | 4,816 (23%) | 942 (20%) | 139 (20%) |
| Second | 68,585 (27%) | 17,809 (28%) | 5,962 (28%) | 1,271 (27%) | 181 (25%) |
| Third | 49,632 (19%) | 12,244 (19%) | 4,145 (19%) | 952 (20%) | 152 (21%) |
| Fourth | 39,622 (16%) | 9,928 (16%) | 3,459 (16%) | 856 (18%) | 122 (17%) |
| Fifth (most deprived) | 31,352 (12%) | 7,996 (12%) | 2,918 (14%) | 694 (15%) | 114 (16%) |
| Region |  |  |  |  |  |
| East Midlands | 4,067 (2%) | 1,003 (2%) | 373 (2%) | 68 (1%) | 14 (2%) |
| East of England | 28,423 (11%) | 8,465 (13%) | 2,647 (12%) | 559 (12%) | 72 (10%) |
| London | 31,371 (12%) | 7,524 (12%) | 2,392 (11%) | 522 (11%) | 81 (11%) |
| North East | 3,444 (1%) | 953 (1%) | 296 (1%) | 64 (1%) | 7 (1%) |
| North West | 58,137 (23%) | 14,561 (23%) | 4,629 (22%) | 1,009 (21%) | 137 (19%) |
| South Central | 29,178 (11%) | 7,322 (11%) | 2,633 (12%) | 629 (13%) | 95 (13%) |
| South East Coast | 21,037 (8%) | 4,092 (6%) | 1,499 (7%) | 337 (7%) | 55 (8%) |
| South West | 33,649 (13%) | 7,855 (12%) | 2,920 (14%) | 662 (14%) | 107 (15%) |
| West Midlands | 40,702 (16%) | 10,678 (17%) | 3,475 (16%) | 775 (16%) | 127 (18%) |
| Yorkshire & The Humber | 5,460 (2%) | 1,554 (2%) | 504 (2%) | 105 (2%) | 15 (2%) |

BMI, body mass index; CKD, chronic kidney disease; CPRD, Clinical Practice Research Datalink; eGFR, estimated glomerular filtration rate; HDL, high-density lipoprotein; LDL, low-density lipoprotein; RRT, renal replacement therapy.

Values presented are means (SD) or number (percentage).

^1^ Defined as having had at least 2 prescriptions by the cohort entry date, with at least one prescription ≤84 days before the cohort entry

## **Table E. Study participants (%) experiencing adverse events of interest during follow-up, by eGFR at cohort entry**

|  | **eGFR 60-89 ml/min/1.73m^2^**  **(G2)** | **eGFR 45-59**  **ml/min/1.73m^2^**  **(G3a)** | **eGFR 30-44**  **ml/min/1.73m^2^**  **(G3b)** | **eGFR 15-29**  **ml/min/1.73m^2^**  **(G4)** | **eGFR <15**  **ml/min/1.73m^2^**    **(G5) not on RRT** |
| --- | --- | --- | --- | --- | --- |
| **Estimation cohort** |  |  |  |  |  |
| **N** | **592,179** | **142,051** | **46,724** | **10,495** | **1,816** |
| Nonvascular death | 36,088 (6%) | 21,935 (15%) | 12,124 (26%) | 3,649 (35%) | 706 (39%) |
| Vascular death | 10,930 (2%) | 8,667 (6%) | 5,604 (12%) | 1,607 (15%) | 220 (12%) |
| Vascular death or stroke | 22,520 (4%) | 14,932 (11%) | 8,114 (17%) | 2,100 (20%) | 306 (17%) |
| Vascular death, stroke or MI | 32,523 (5%) | 19,425 (14%) | 10,110 (22%) | 2,594 (25%) | 396 (22%) |
| Heart failure hospitalisation | 18,713 (3%) | 13,676 (10%) | 8,570 (18%) | 2,768 (26%) | 347 (19%) |
| **Validation cohort** |  |  |  |  |  |
| **N** | **225,468** | **64,007** | **21,368** | **4,730** | **710** |
| Nonvascular death | 15,527 (6%) | 9,748 (15%) | 5,620 (26%) | 1,629 (34%) | 272 (38%) |
| Vascular death | 4,755 (2%) | 3,956 (6%) | 2,644 (12%) | 750 (16%) | 81 (11%) |
| Vascular death or stroke | 9,784 (4%) | 6,636 (10%) | 3,807 (18%) | 978 (21%) | 106 (15%) |
| Vascular death, stroke or MI | 14,103 (6%) | 8,625 (13%) | 4,736 (22%) | 1,215 (26%) | 146 (21%) |
| Heart failure hospitalisation | 8,355 (8%) | 6,243 (10%) | 3,993 (19%) | 1,250 (26%) | 177 (25%) |

eGFR, estimated glomerular filtration rate; MI, myocardial infarction; RRT, renal replacement therapy

## **Table F. Risk equations for nonvascular death among patients with reduced kidney function, CPRD estimation cohort**

|  |  | **Females without prior CVD** | **Females with prior CVD** | **Males without prior CVD** | **Males with prior CVD** |
| --- | --- | --- | --- | --- | --- |
| **Covariates** | **Category** |  |  |  |  |
|  | | Gompertz PH  HR (95% CI) | Gompertz PH  HR (95% CI) | Gompertz PH  HR (95% CI) | Gompertz PH  HR (95% CI) |
| **Patient’s characteristics at cohort entry** | | | | | |
| Albuminuria status (ref: not measured) | Normoalbuminuria | 0.82 (0.80, 0.85) | 0.75 (0.72, 0.77) | 0.87 (0.84, 0.89) | 0.84 (0.81, 0.87) |
|  | Microalbuminuria | 1.29 (1.19, 1.40) | 1.08 (0.98, 1.19) | 1.20 (1.11, 1.30) | 1.11 (1.01, 1.21) |
|  | Macroalbuminuria | 1.53 (1.33, 1.76) | 1.40 (1.20, 1.63) | 1.25 (1.08, 1.44) | 1.28 (1.12, 1.45) |
| **Characteristics updated annually** | | | | | |
| Age | Per 10 years older | 2.49 (2.46, 2.52) | 2.25 (2.21, 2.30) | 2.62 (2.59, 2.66) | 2.36 (2.31, 2.41) |
| eGFR category at the end of the previous year  (ref: eGFR 60-89 ml/min/1.73m2 [G2]) | eGFR 45-59  ml/min/1.73m^2^ (G3a) | 0.98 (0.95, 1.01) | 0.89 (0.85, 0.93) | 0.97 (0.94, 1.00) | 0.93 (0.89, 0.98) |
|  | eGFR 30-44  ml/min/1.73m^2^ (G3b) | 1.24 (1.20, 1.29) | 1.01 (0.96, 1.06) | 1.34 (1.29, 1.40) | 1.25 (1.19, 1.31) |
|  | eGFR 15-29  ml/min/1.73m^2^ (G4) | 2.06 (1.95, 2.17) | 1.57 (1.47, 1.67) | 2.14 (2.01, 2.28) | 1.87 (1.75, 2.00) |
|  | eGFR <15  ml/min/1.73m^2^ (G5) or RRT | 5.82 (5.18, 6.55) | 4.10 (3.62, 4.65) | 4.94 (4.42, 5.52) | 4.17 (3.72, 4.67) |
| Intercept |  | -5.065  (-5.095, -5.036) | -4.242  (-4.292, -4.193) | -4.836  (-4.867, -4.805) | -4.257  (-4.301, -4.212) |
| Ancillary parameter |  | -0.017  (-0.021, -0.012) | -0.030  (-0.037, -0.023) | -0.028  (-0.033, -0.023) | -0.040  (-0.047, -0.033) |

CI, confidence interval; CPRD, Clinical Practice Research Datalink; CVD, cardiovascular disease; eGFR, Estimated Glomerular Filtration Rate; HR, hazard ratio; PH, proportional hazards; RRT, renal replacement therapy

Each risk equation included further adjustments for use of lipid-lowering; antihypertensive and antiplatelet therapies. The intercept and ancillary parameters are presented on the original scale.

## **Table G. Risk equations for heart failure hospital admission among patients with reduced kidney function, CPRD estimation cohort**

| **Covariates** | **Category** | **Females without prior CVD** | **Females with prior cCVD** | **Males without prior CVD** | **Males with prior CVD** |
| --- | --- | --- | --- | --- | --- |
|  | | Weibull PH  HR (95% CI) | Weibull PH  HR (95% CI) | Weibull PH  HR (95% CI) | Weibull PH  HR (95% CI) |
| **Patient’s characteristics at cohort entry** | | | | | |
| *Sociodemographic characteristics* | | | | | |
| Smoker  (ref: never smoked) | Former | 1.25 (1.19, 1.30) | 1.16 (1.11, 1.21) | 1.15 (1.10, 1.21) | 1.13 (1.08, 1.17) |
|  | Current | 1.62 (1.53, 1.71) | 1.36 (1.27, 1.45) | 1.60 (1.51, 1.69) | 1.26 (1.19, 1.33) |
| BMI  (ref: ≥18.5, <25 kg/m^2^) | <18.5 kg/m^2^ | 1.10 (0.96, 1.25) | 0.99 (0.89, 1.11) | 1.15 (0.94, 1.40) | 0.95 (0.79, 1.14) |
|  | ≥25, <30 kg/m^2^ | 1.10 (1.04, 1.15) | 1.04 (0.99, 1.09) | 1.14 (1.08, 1.20) | 1.06 (1.01, 1.12) |
|  | ≥30, <35 kg/m^2^ | 1.41 (1.34, 1.49) | 1.19 (1.12, 1.27) | 1.48 (1.38, 1.59) | 1.24 (1.17, 1.32) |
|  | ≥35, <40 kg/m^2^ | 1.94 (1.80, 2.10) | 1.43 (1.30, 1.56) | 2.30 (2.08, 2.54) | 1.57 (1.44, 1.70) |
|  | ≥40 kg/m^2^ | 3.02 (2.75, 3.33) | 2.02 (1.82, 2.25) | 3.06 (2.64, 3.56) | 1.92 (1.69, 2.17) |
| Index of multiple deprivation quintile  (ref: First quintile:least deprived) | Second quintile | 1.05 (1.00, 1.12) | 1.07 (1.01, 1.14) | 1.06 (0.99, 1.12) | 1.06 (1.00, 1.12) |
|  | Third quintile | 1.16 (1.10, 1.23) | 1.11 (1.04, 1.18) | 1.16 (1.09, 1.24) | 1.11 (1.05, 1.17) |
|  | Fourth quintile | 1.29 (1.21, 1.36) | 1.19 (1.12, 1.27) | 1.28 (1.20, 1.36) | 1.21 (1.15, 1.29) |
|  | Fifth quintile (most deprived) | 1.42 (1.33, 1.51) | 1.29 (1.21, 1.37) | 1.35 (1.26, 1.45) | 1.24 (1.17, 1.32) |
| *Disease history, laboratory measurements and other risk factors* | | | | | |
| Diabetes  (ref: no diabetes) | Type I | 2.35 (1.62, 3.42) | 1.96 (1.45, 2.65) | 1.69 (1.17, 2.42) | 2.04 (1.65, 2.51) |
|  | Type II | 1.41 (1.34, 1.49) | 1.26 (1.20, 1.33) | 1.20 (1.14, 1.28) | 1.30 (1.24, 1.36) |
| Albuminuria status (ref: not measured) | Normoalbuminuria | 0.89 (0.85, 0.92) | 0.94 (0.90, 0.98) | 0.95 (0.90, 0.99) | 0.97 (0.93, 1.01) |
|  | Microalbuminuria | 1.09 (0.97, 1.23) | 1.00 (0.90, 1.11) | 1.26 (1.12, 1.41) | 1.10 (1.00, 1.20) |
|  | Macroalbuminuria | 1.53 (1.27, 1.85) | 1.17 (0.99, 1.37) | 1.42 (1.18, 1.70) | 1.12 (0.98, 1.27) |
| Total cholesterol : HDL cholesterol ratio | Per unit increase | 0.94 (0.92, 0.95) | 0.94 (0.90, 0.98) | 0.93 (0.92, 0.95) | 0.95 (0.94, 0.97) |
| Systolic Blood Pressure  Centred at 139 mmHg | Per 20 mmHg increase | 1.06 (1.04, 1.08) | 1.00 (0.98, 1.02) | 1.02 (1.00, 1.04) | 0.93 (0.91, 0.95) |
| Rheumatoid arthritis | Yes | 1.68 (1.53, 1.85) | 1.26 (1.14, 1.40) | 1.54 (1.32, 1.81) | 1.51 (1.34, 1.71) |
| Atrial fibrillation | Yes | 2.41 (2.25, 2.58) | 1.41 (1.35, 1.47) | 2.14 (1.99, 2.31) | 1.42 (1.36, 1.48) |
| Diagnosis of mental illness | Yes | 1.22 (1.13, 1.33) | 1.07 (0.99, 1.17) | 1.11 (0.98, 1.26) | 1.11 (1.00, 1.22) |
| Family history of coronary heart disease | Yes | 0.97 (0.92, 1.03) | N/A | 1.06 (1.00, 1.13) | N/A |
| History of coronary heart disease | Yes | N/A | 1.50 (1.42, 1.59) | N/A | 1.42 (1.35, 1.51) |
| History of cerebrovascular disease | Yes |  | 0.99 (0.95, 1.04) |  | 1.12 (1.08, 1.17) |
| History of heart failure | Yes |  | 3.33 (3.19, 3.48) |  | 3.60 (3.46, 3.74) |
| **Characteristics updated annually** | | | | | |
| Age | Per 10 years older | 2.46 (2.40, 2.51) | 1.48 (1.44, 1.51) | 2.25 (2.20, 2.31) | 1.45 (1.42, 1.48) |
| eGFR category at the end of the previous year  (ref: eGFR 60-89 ml/min/1.73m2 [G2]) | eGFR 45-59  ml/min/1.73m^2^ (G3a) | 1.22 (1.17, 1.28) | 1.29 (1.22, 1.36) | 1.31 (1.24, 1.38) | 1.35 (1.29, 1.41) |
|  | eGFR 30-44  ml/min/1.73m^2^ (G3b) | 1.59 (1.50, 1.67) | 1.61 (1.53, 1.70) | 1.72 (1.61, 1.83) | 1.75 (1.66, 1.84) |
|  | eGFR 15-29  ml/min/1.73m^2^ (G4) | 2.54 (2.36, 2.73) | 2.14 (1.99, 2.29) | 2.94 (2.69, 3.22) | 2.39 (2.23, 2.57) |
|  | eGFR <15  ml/min/1.73m^2^ (G5) or RRT | 3.57 (2.91, 4.37) | 2.53 (2.13, 3.00) | 4.10 (3.43, 4.89) | 2.25 (1.94, 2.61) |
| Cardiovascular event during follow-up  (ref: no MI or stroke during follow-up) | MI | 29.55 (27.31, 31.97) | 10.07 (9.20, 11.03) | 31.22 (29.01, 33.60) | 9.77 (9.05, 10.55) |
|  | Stroke | 6.22 (5.98, 6.46) | 5.01 (4.81, 5.21) | 5.77 (5.53, 6.03) | 4.71 (4.53, 4.89) |
| Intercept |  | -7.100  (-7.198, -7.003) | -5.258  (-5.370, -5.146) | -6.640  (-6.748, -6.533) | -5.139  (-5.253, -5.024) |
| Ancillary parameter |  | -0.012  (-0.029, 0.005) | -0.084  (-0.099, -0.069) | -0.032  (-0.050, -0.013) | -0.097  (-0.112, -0.082) |

BMI, body mass index; CI, confidence interval; CPRD, Clinical Practice Research Datalink; CVD, cardiovascular disease; eGFR, estimated glomerular filtration rate; HDL, high density lipoprotein; HR, hazard ratio; MI, Myocardial infarction; N/A, not applicable: covariate not included or specified through other covariates within category; PH, proportional hazards.; RRT, renal replacement therapy.

Each risk equation included further adjustments for use of lipid-lowering; antihypertensive and antiplatelet therapies. The intercept and ancillary parameters are presented on the original scale.

## **Fig B. Comparison of cumulative risks predicted by the model and observed Kaplan-Meier risks in the validation cohort, for nonvascular death and first heart failure hospitalisation and by eGFR category at cohort entry**


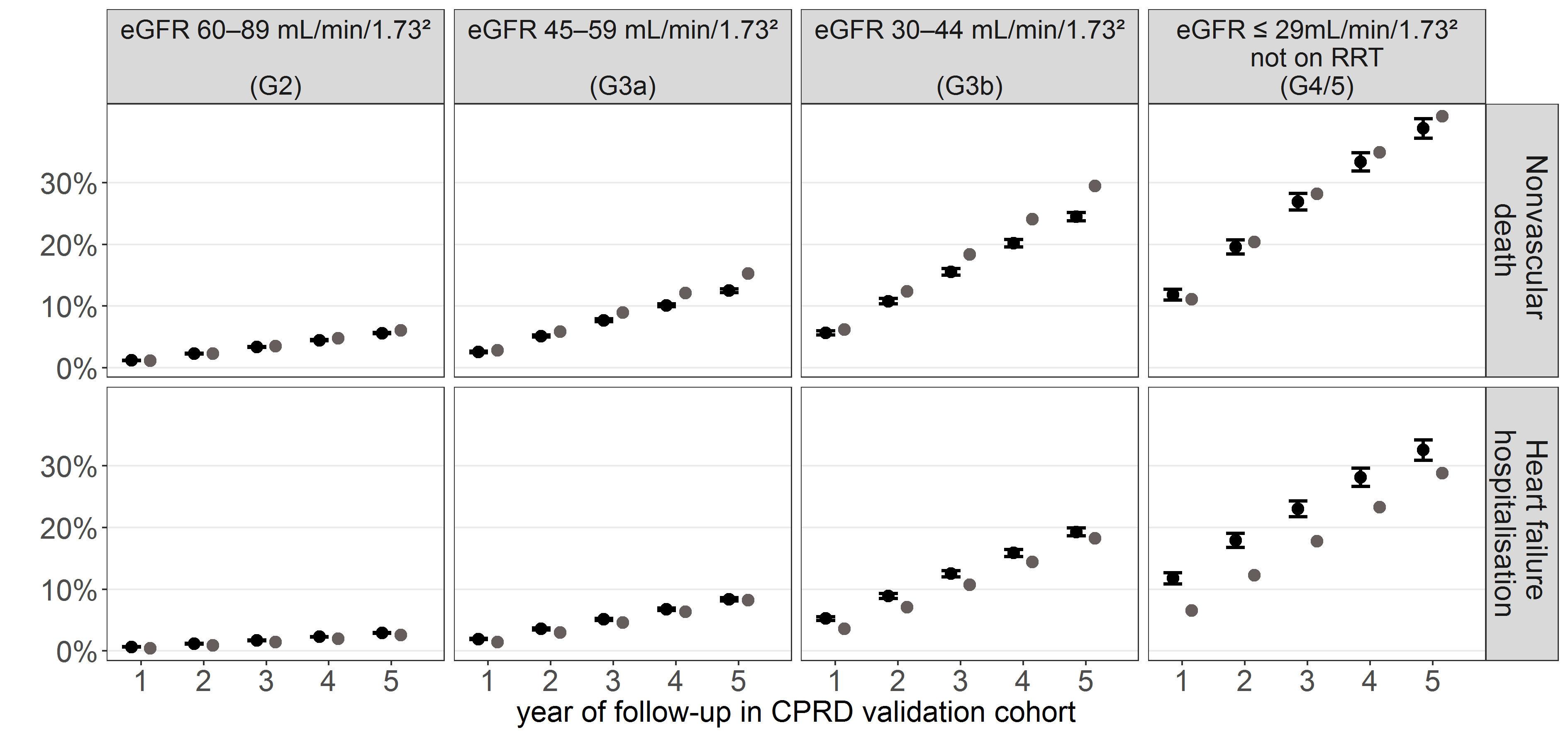


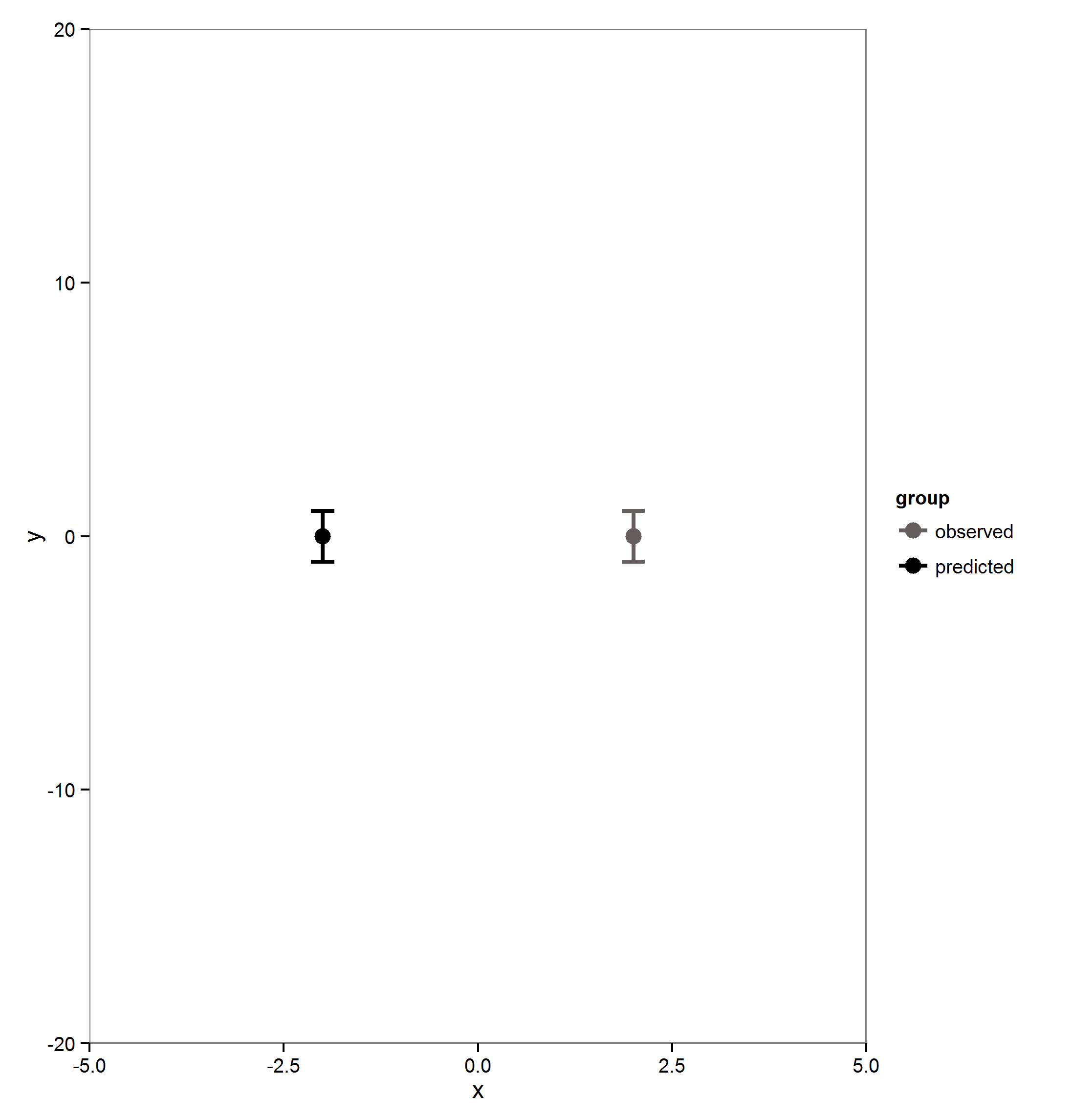


Observed (95% CI)

Predicted by the CKD-CVD model


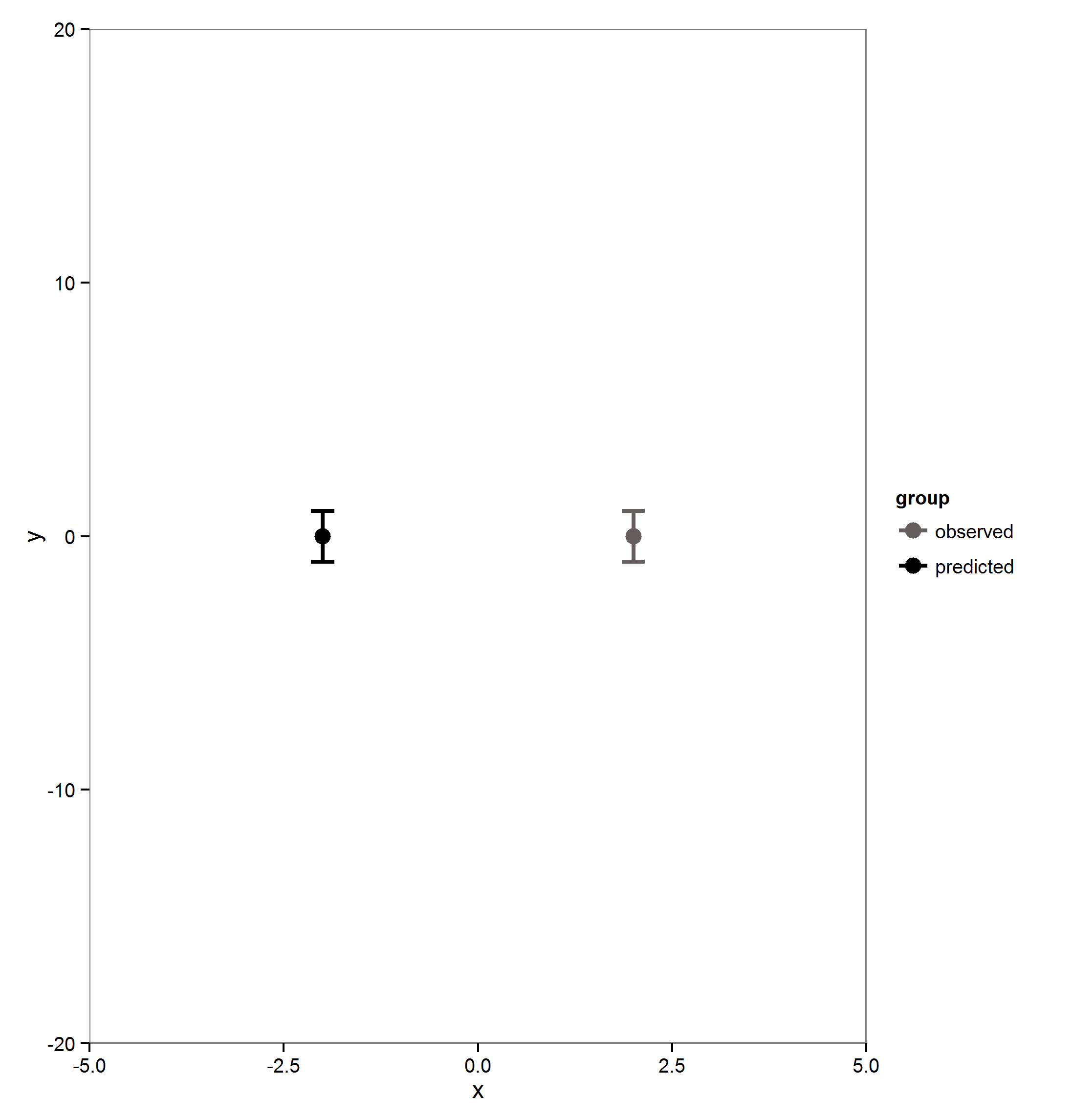


Footnote: CI, confidence interval; CKD, chronic kidney disease; CPRD, Clinical Practice Research Datalink; CVD, cardiovascular disease; eGFR, estimated glomerular filtration rate; RRT, renal replacement therapy.

## **Fig C. Comparison of cumulative risks predicted by the model and observed Kaplan-Meier risks in the validation cohort, for the combined endpoint of vascular death, stroke or myocardial infarction, by eGFR category at cohort entry and practice location**

1. North West, Nort East and Yorkshire & The Humber


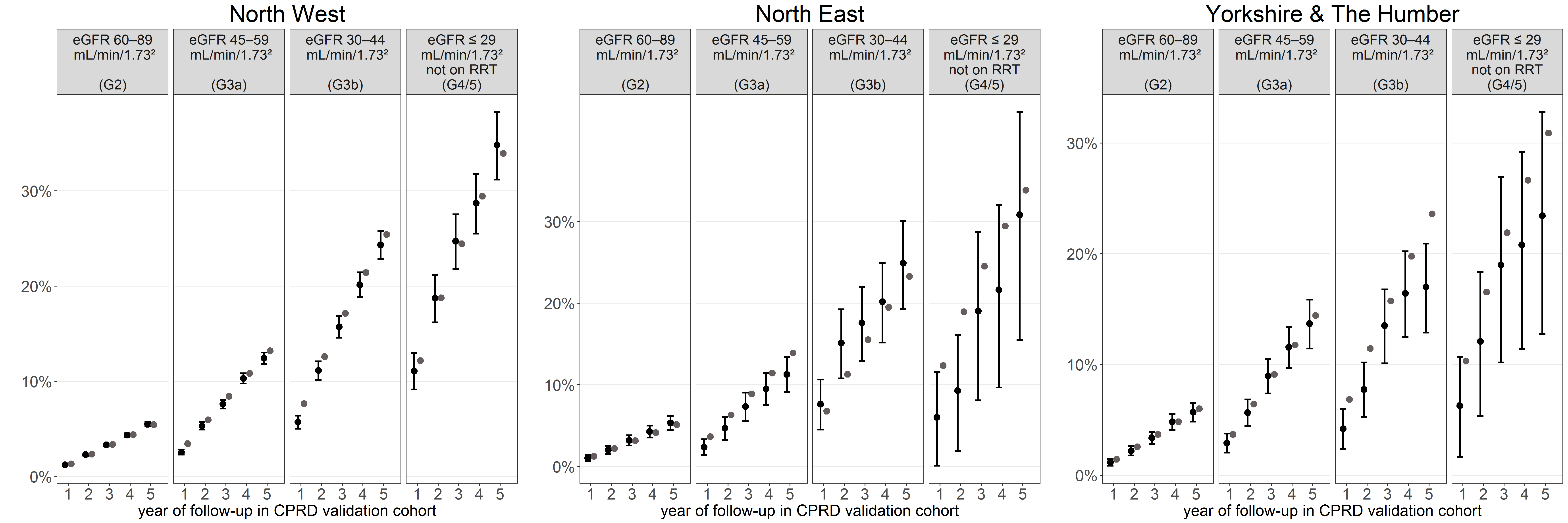


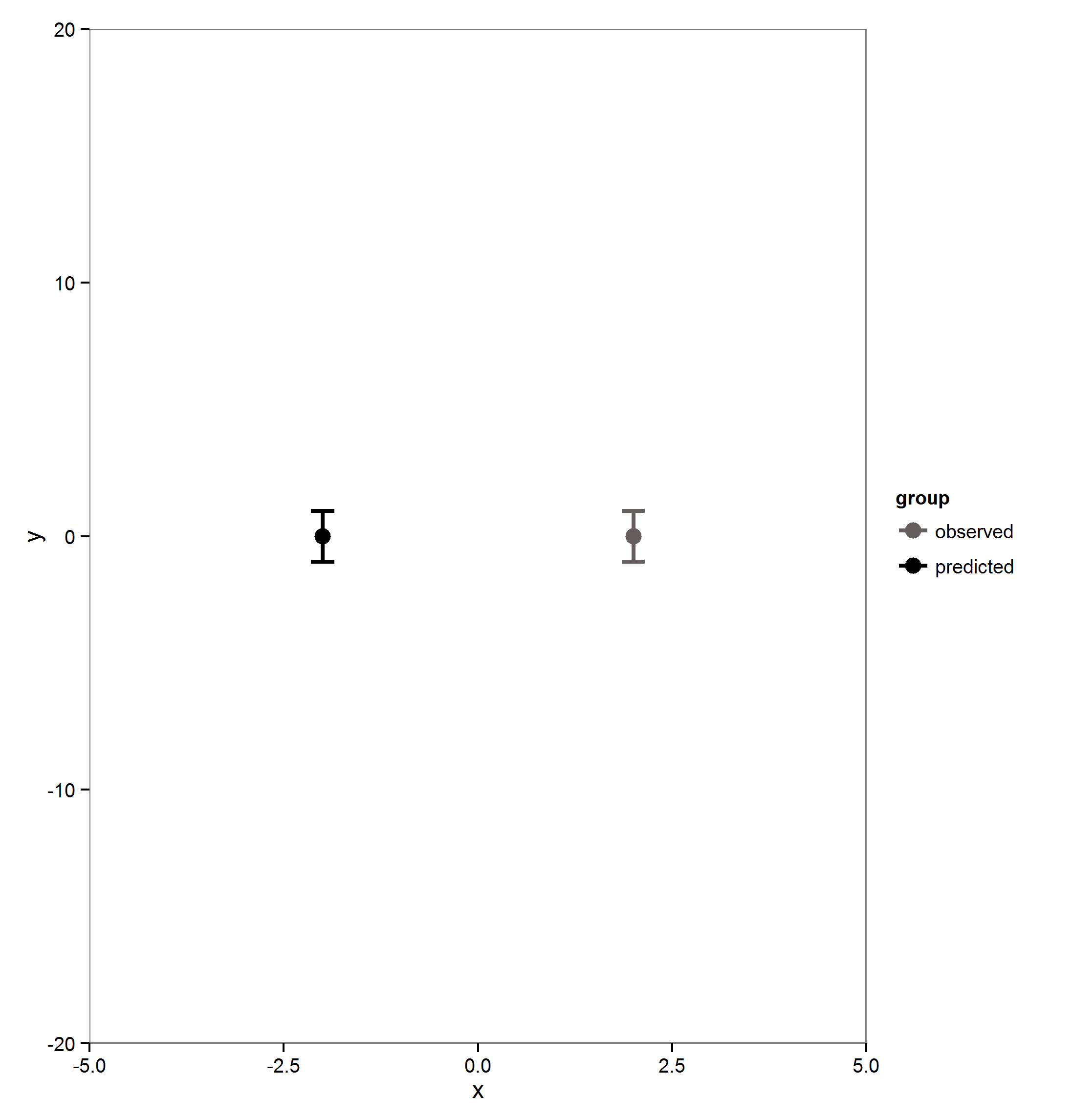


Observed (95% CI)

Predicted by the CKD-CVD model


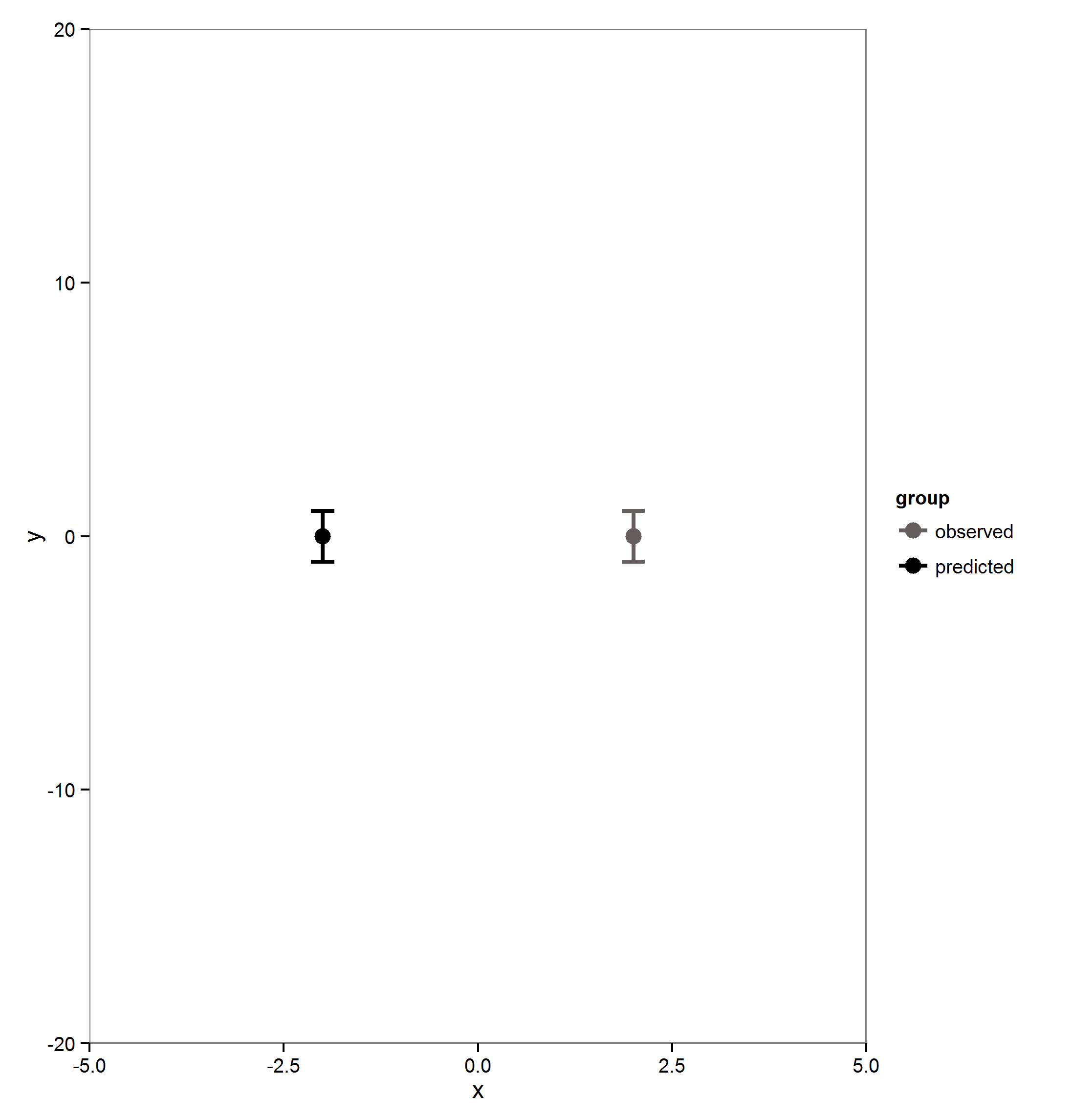


1. West Midlands, East Midlands and East of England


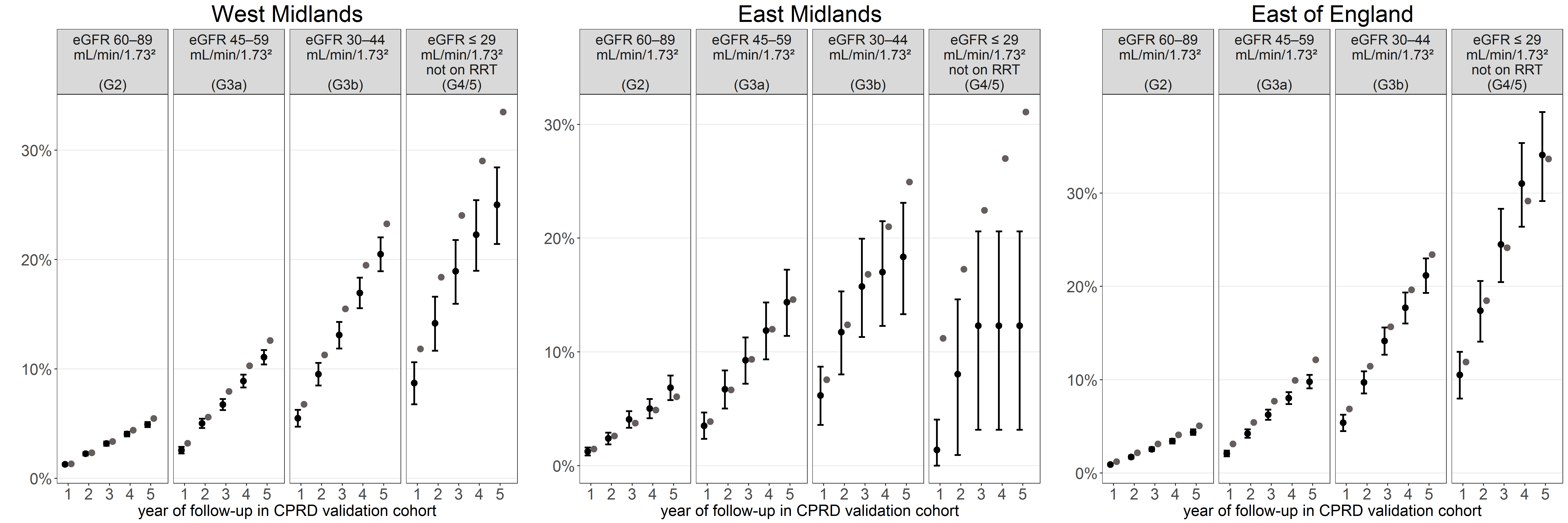


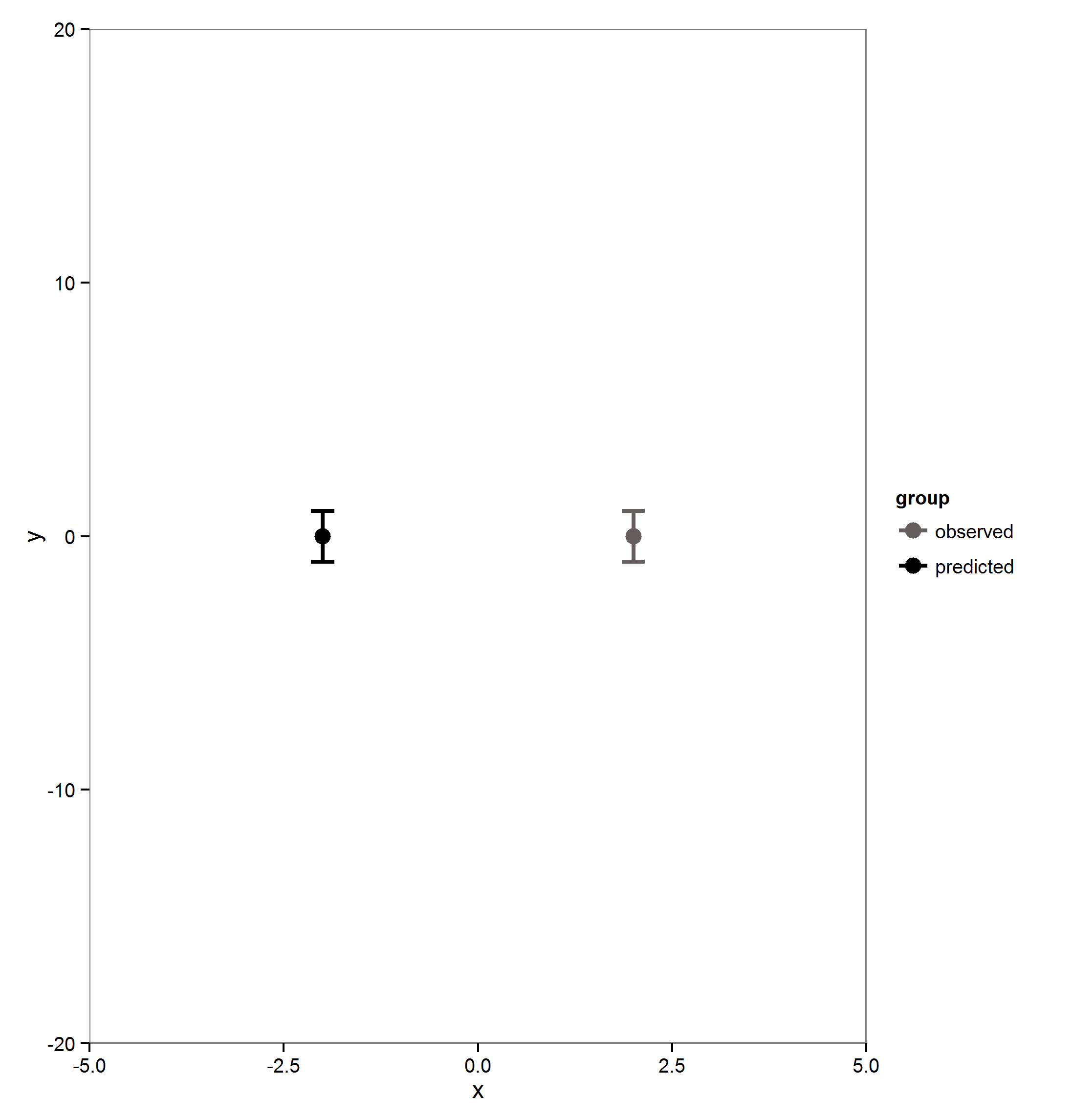


Observed (95% CI)

Predicted by the CKD-CVD model


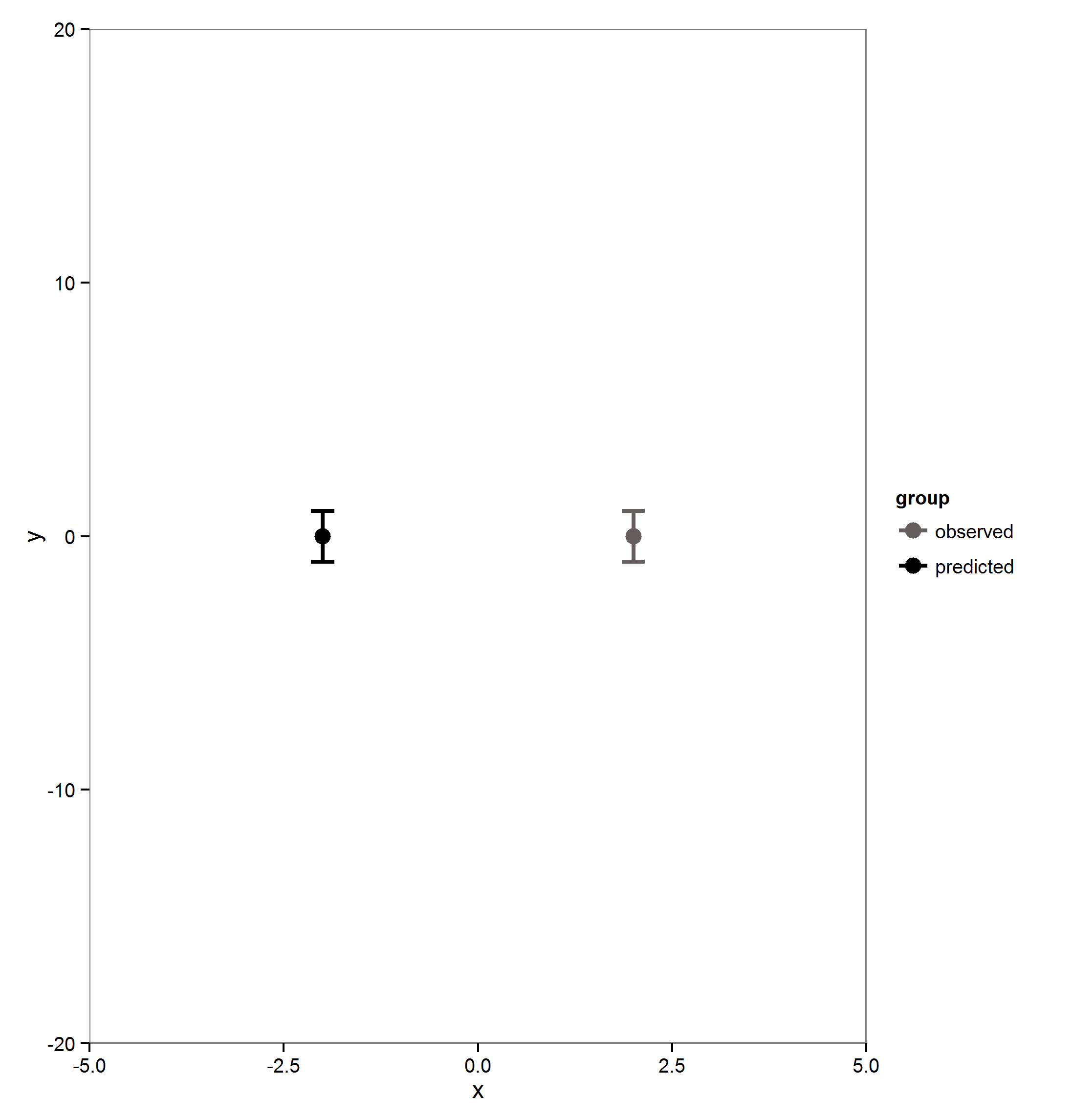


**
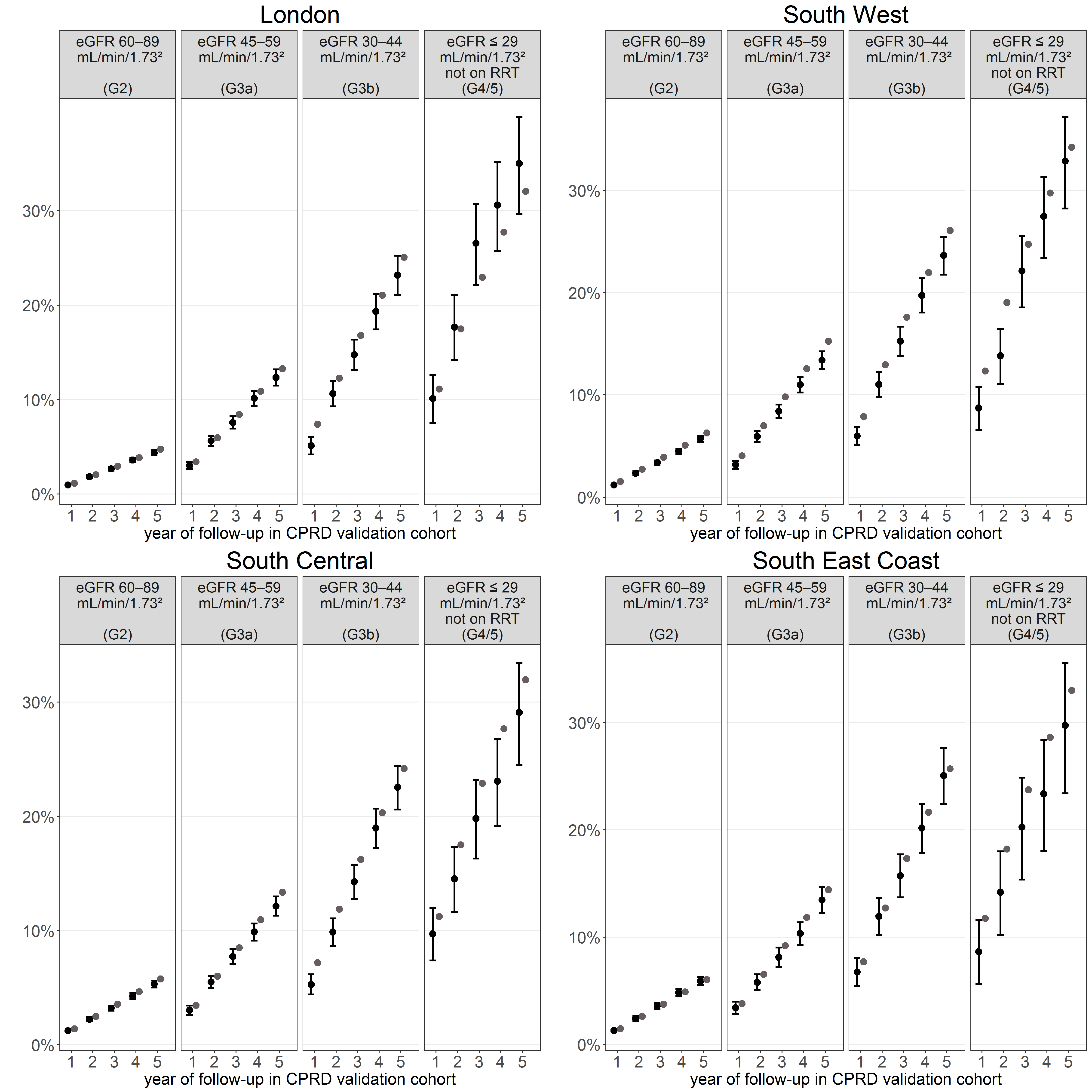
**


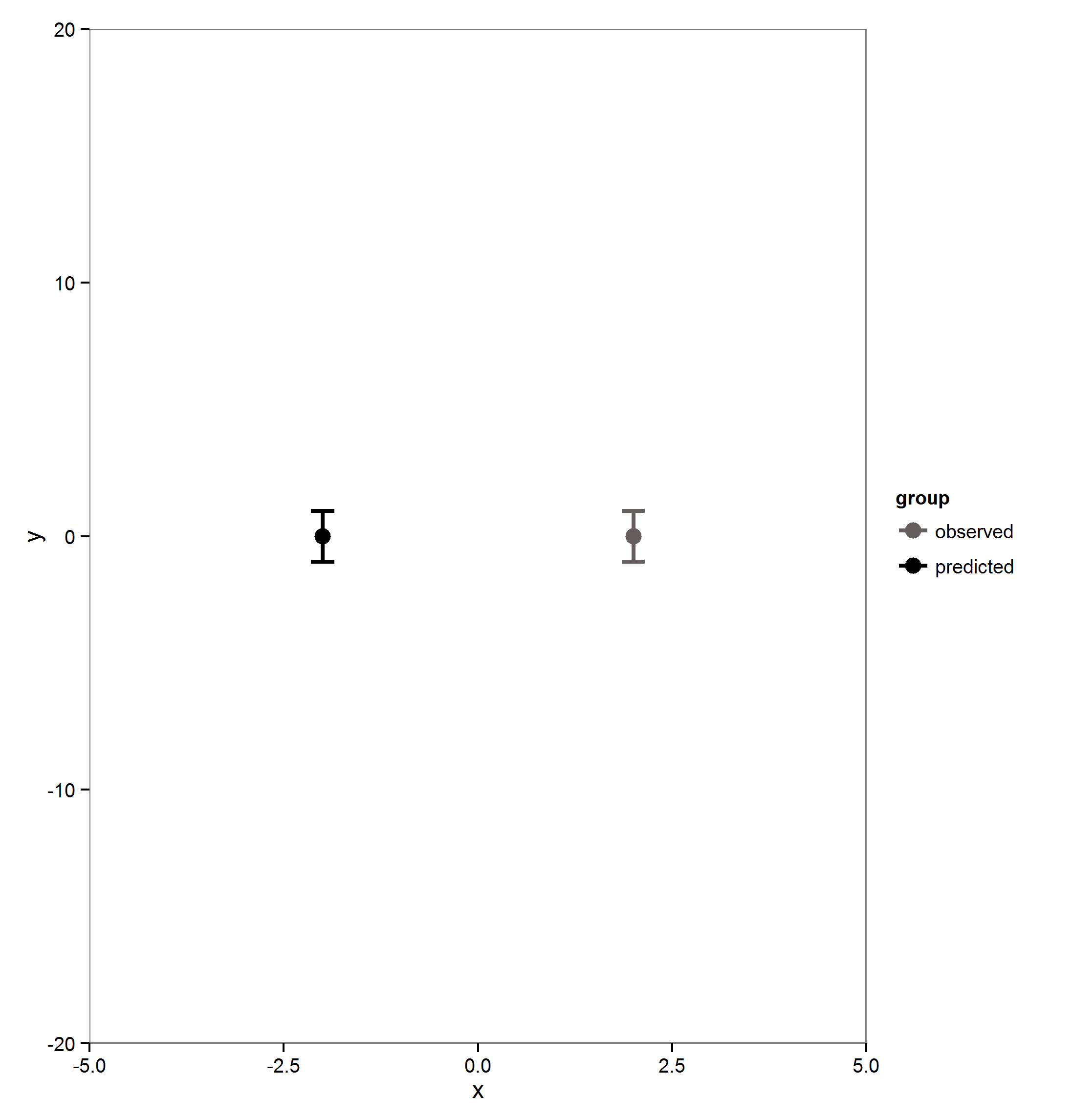


Observed (95% CI)

Predicted by the CKD-CVD model


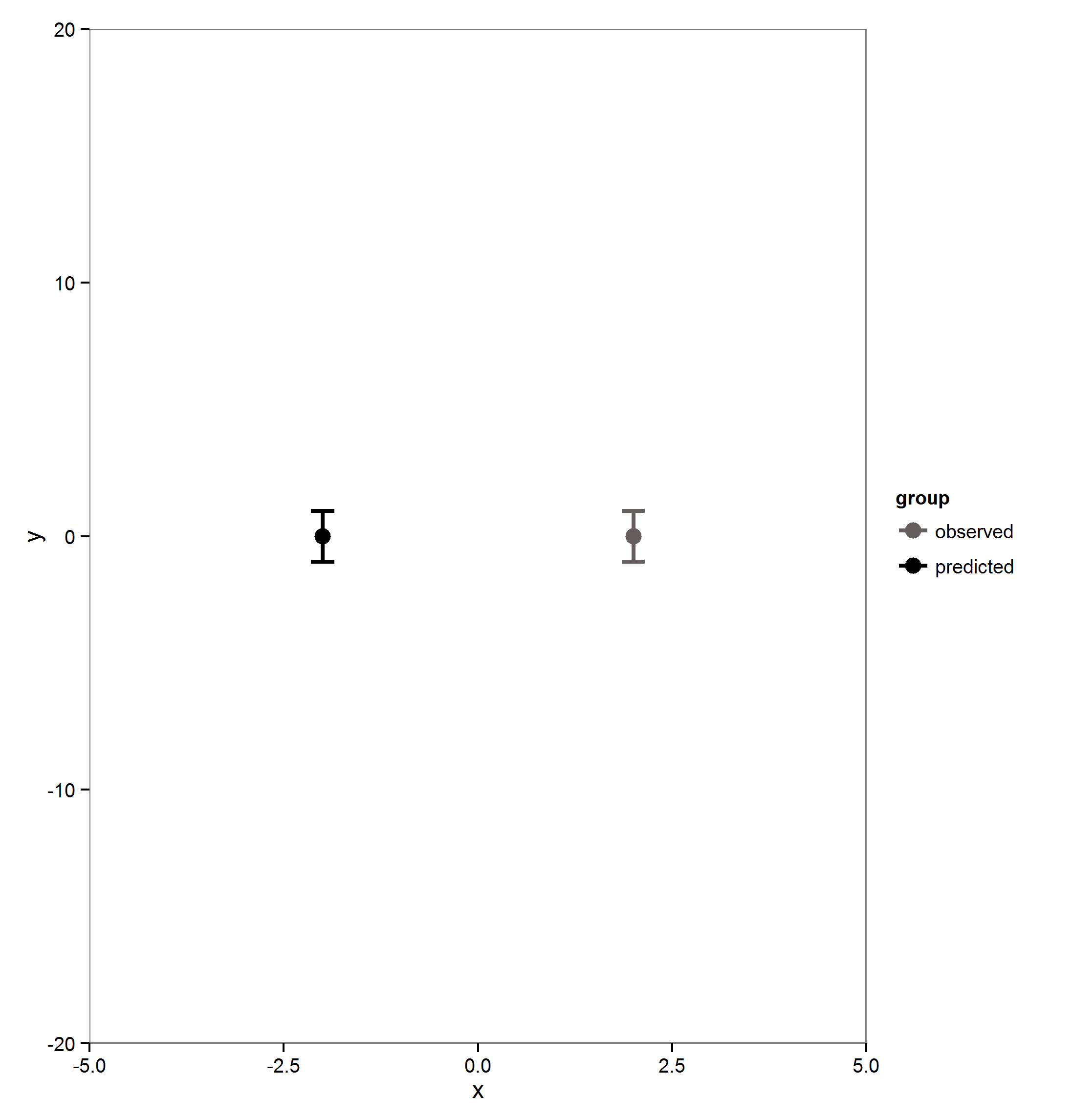


1. London, South West, South Central and South East Coast

Footnote: eGFR, estimated glomerular filtration rate; CI, confidence interval; CKD, chronic kidney disease; CPRD, Clinical Practice Research Datalink; CVD, cardiovascular disease; RRT, renal replacement therapy

## **Table H. Harrel’s C-index (95% confidence intervals) assessing the discrimination of the CKD-CVD policy model at 5 years, by patient’s eGFR stage at cohort entry**

| eGFR stage at cohort entry | **Vascular death** | **Vascular death or stroke** | **Vascular death, stroke or MI** |
| --- | --- | --- | --- |
| ***Estimation cohort*** | | | |
| eGFR 60-89  ml/min/1.73m^2^ (G2) | 0.84 (0.83, 0.84) | 0.81 (0.81, 0.81) | 0.78 (0.78, 0.79) |
| eGFR 45-59  ml/min/1.73m^2^ (G3a) | 0.75 (0.74, 0.76) | 0.75 (0.74, 0.75) | 0.73 (0.73, 0.73) |
| eGFR 30-44  ml/min/1.73m^2^ (G3b) | 0.66 (0.65, 0.67) | 0.69 (0.68, 0.69) | 0.67 (0.67, 0.68) |
| eGFR 15-29  ml/min/1.73m^2^ (G4) | 0.60 (0.59, 0.62) | 0.66 (0.65, 0.68) | 0.65 (0.64, 0.66) |
| eGFR <15  ml/min/1.73m^2^ (G5) not on RRT | 0.65 (0.61, 0.69) | 0.70 (0.67, 0.73) | 0.69 (0.67, 0.72) |
| ***Validation cohort*** | | | |
| eGFR 60-89  ml/min/1.73m^2^ (G2) | 0.84 (0.83, 0.85) | 0.82 (0.81, 0.82) | 0.79 (0.79, 0.79) |
| eGFR 45-59  ml/min/1.73m^2^ (G3a) | 0.75 (0.75, 0.76) | 0.75 (0.75, 0.76) | 0.73 (0.73, 0.74) |
| eGFR 30-44  ml/min/1.73m^2^ (G3b) | 0.66 (0.65, 0.68) | 0.69 (0.68, 0.70) | 0.68 (0.67, 0.68) |
| eGFR 15-29  ml/min/1.73m^2^ (G4) | 0.62 (0.59, 0.64) | 0.66 (0.65, 0.68) | 0.66 (0.64, 0.67) |
| eGFR <15  ml/min/1.73m^2^ (G5) not on RRT | 0.66 (0.59, 0.73) | 0.68 (0.63, 0.73) | 0.66 (0.62, 0.71) |

CKD, chronic kidney disease; CVD, cardiovascular disease; eGFR, estimated glomerular filtration rate; MI, myocardial infarction; RRT, renal replacement therapy.

The Harrell’s C-index is a non-parametric rank-correlation measure used for a censored response. A value above 0.5 indicates positive predictive discrimination. The C-index was calculated at the median follow-up time in the cohort of five years.

## **Table I. Predicted life expectancy and quality-adjusted life years without cardiovascular prevention treatments, by CVD history, age and eGFR category at cohort entry**

| **eGFR stage at**  **cohort entry** | **Age at cohort entry, years** | | | | | | | |
| --- | --- | --- | --- | --- | --- | --- | --- | --- |
|  | **Without CVD** | | | | **With CVD** | | | |
|  | **<60** | **60-69** | **70-79** | **≥80** | **<60** | **60-69** | **70-79** | **≥80** |
| **Predicted life expectancy, years (95% confidence intervals)** | | | | | | | | |
| eGFR 60-89 mL/min/1.73^2^ (G2) | 38.1  (37.5 - 38.6) | 22.1  (21.8 - 22.3) | 14.1  (14.0 - 14.2) | 7.8  (7.7 - 7.9) | 32.4  (31.6 - 33.1) | 18.6  (18.2 - 18.9) | 11.4  (11.3 - 11.6) | 6.0  (5.9 - 6.1) |
| eGFR 45-59  ml/min/1.73m^2^ (G3a) | 32.9  (32.4 - 33.3) | 20.7  (20.4 - 20.9) | 13.0  (12.9 - 13.2) | 7.1  (7.0 - 7.2) | 28.2  (27.4 - 28.8) | 17.3  (16.9 - 17.7) | 10.6  (10.4 - 10.8) | 5.7  (5.6 - 5.7) |
| eGFR 30-44  ml/min/1.73m^2^ (G3b) | 33.3  (32.6 - 33.9) | 18.4  (18.1 - 18.6) | 11.4  (11.3 - 11.5) | 6.0  (6.0 - 6.1) | 25.8  (25.0 - 26.4) | 14.9  (14.6 - 15.2) | 9.1  (9.0 - 9.3) | 4.9  (4.8 - 4.9) |
| eGFR 15-29  ml/min/1.73m^2^ (G4) | 32.7  (31.9 - 33.3) | 16.0  (15.7 - 16.3) | 9.8  (9.6 - 9.9) | 4.8  (4.7 - 4.9) | 22.4  (21.7 - 23.1) | 12.7  (12.3 - 13.0) | 7.8  (7.6 - 7.9) | 4.0  (4.0 - 4.1) |
| eGFR <15  ml/min/1.73m^2^ (G5), not on RRT | 29.3  (28.4 - 30.2) | 13.7  (13.2 - 14.2) | 8.5  (8.2 - 8.8) | 3.7  (3.6 - 3.8) | 20.5  (19.6 - 21.4) | 10.1  (9.7 - 10.6) | 6.5  (6.2 - 6.7) | 3.3  (3.2 - 3.4) |
| **Predicted quality-adjusted life expectancy, QALYs (95% confidence intervals)** | | | | | | | | |
| eGFR 60-89 mL/min/1.73^2^ (G2) | 28.6  (27.8 - 29.3) | 16.1  (15.6 - 16.5) | 10.1  (9.8 - 10.4) | 5.6  (5.4 - 5.7) | 20.6  (19.7 - 21.5) | 11.8  (11.2 - 12.3) | 7.2  (6.9 - 7.5) | 3.7  (3.6 - 3.9) |
| eGFR 45-59  ml/min/1.73m^2^ (G3a) | 24.1  (23.4 - 24.9) | 14.8  (14.3 - 15.2) | 9.2  (8.9 - 9.5) | 5.0  (4.8 - 5.1) | 17.2  (16.4 - 18.0) | 10.7  (10.2 - 11.2) | 6.5  (6.2 - 6.8) | 3.4  (3.3 - 3.6) |
| eGFR 30-44  ml/min/1.73m^2^ (G3b) | 23.5  (22.7 - 24.3) | 12.5  (12.1 - 13.0) | 7.8  (7.5 - 8.0) | 4.1  (3.9 - 4.2) | 14.7  (13.9 - 15.5) | 8.5  (8.0 - 9.0) | 5.3  (5.0 - 5.6) | 2.8  (2.6 - 2.9) |
| eGFR 15-29  ml/min/1.73m^2^ (G4) | 22.6  (21.6 - 23.7) | 10.6  (10.1 - 11.1) | 6.6  (6.3 - 6.9) | 3.2  (3.0 - 3.3) | 12.6  (11.7 - 13.4) | 6.9  (6.5 - 7.4) | 4.3  (4.0 - 4.6) | 2.2  (2.0 - 2.3) |
| eGFR <15  ml/min/1.73m^2^ (G5) not on RRT | 20.5  (19.7 - 21.4) | 9.2  (8.7 - 9.7) | 5.7  (5.4 - 6.0) | 2.4  (2.3 - 2.5) | 11.6  (10.8 - 12.4) | 5.7  (5.3 - 6.2) | 3.6  (3.4 - 3.9) | 1.8  (1.7 - 2.0) |

CVD, cardiovascular disease; eGFR, estimated glomerular filtration rate; RRT, renal replacement therapy;

The predictions are based on a random sample of 64,000 patients from the whole cohort, see Methods section for further details of how the sampling was performed.

## **Table J. Predicted life years and quality-adjusted life years gained (a) with 2013 cardiovascular prevention medications use compared to no use, and (b) additional gains with optimal medication use, by CVD history, age and eGFR category at cohort entry**

| **eGFR stage at**  **cohort entry** | **Age at cohort entry, years** | | | | | | | |
| --- | --- | --- | --- | --- | --- | --- | --- | --- |
|  | **Without CVD** | | | | **With CVD** | | | |
|  | **<60** | **60-69** | **70-79** | **≥80** | **<60** | **60-69** | **70-79** | **≥80** |
| **(a) Predicted life years gained with 2013 use of statins, antihypertensives and antiplatelets compared to no use**  **Years (95% confidence intervals)** | | | | | | | | |
| eGFR 60-89 mL/min/1.73^2^ (G2) | 0.19  (0.13 - 0.24) | 0.19  (0.14 - 0.23) | 0.16  (0.12 - 0.19) | 0.11  (0.08 - 0.13) | 1.07  (0.59 - 1.45) | 0.90  (0.50 - 1.21) | 0.65  (0.35 - 0.88) | 0.37  (0.19 - 0.52) |
| eGFR 45-59  ml/min/1.73m^2^ (G3a) | 0.22  (0.15 - 0.28) | 0.20  (0.14 - 0.25) | 0.16  (0.11 - 0.20) | 0.10  (0.07 - 0.13) | 1.22  (0.62 - 1.72) | 0.92  (0.46 - 1.29) | 0.64  (0.31 - 0.90) | 0.35  (0.17 - 0.50) |
| eGFR 30-44  ml/min/1.73m^2^ (G3b) | 0.28  (0.18 - 0.36) | 0.22  (0.15 - 0.29) | 0.17  (0.11 - 0.21) | 0.10  (0.06 - 0.12) | 1.50  (0.71 - 2.12) | 1.01  (0.49 - 1.42) | 0.65  (0.31 - 0.91) | 0.32  (0.15 - 0.45) |
| eGFR 15-29  ml/min/1.73m^2^ (G4) | 0.32  (0.21 - 0.42) | 0.25  (0.17 - 0.32) | 0.17  (0.12 - 0.22) | 0.09  (0.06 - 0.12) | 1.47  (0.77 - 2.03) | 1.00  (0.51 - 1.39) | 0.61  (0.30 - 0.85) | 0.28  (0.13 - 0.39) |
| eGFR <15  ml/min/1.73m^2^ (G5), not on RRT | 0.24  (0.14 - 0.33) | 0.22  (0.14 - 0.28) | 0.14  (0.10 - 0.18) | 0.07  (0.05 - 0.09) | 1.49  (0.69 - 2.17) | 0.95  (0.48 - 1.35) | 0.58  (0.29 - 0.83) | 0.24  (0.11 - 0.34) |
| **Predicted quality-adjusted life years gained with 2013 use of statins, antihypertensives and antiplatelets compared to no use**  **QALYs (95% confidence intervals)** | | | | | | | | |
| eGFR 60-89 mL/min/1.73^2^ (G2) | 0.24  (0.20 - 0.28) | 0.22  (0.18 - 0.25) | 0.16  (0.14 - 0.18) | 0.10  (0.08 - 0.11) | 1.13  (0.61 - 1.51) | 0.80  (0.47 - 1.06) | 0.53  (0.32 - 0.70) | 0.29  (0.17 - 0.39) |
| eGFR 45-59  ml/min/1.73m^2^ (G3a) | 0.28  (0.22 - 0.33) | 0.22  (0.17 - 0.26) | 0.16  (0.13 - 0.19) | 0.10  (0.07 - 0.12) | 1.21  (0.65 - 1.60) | 0.82  (0.47 - 1.08) | 0.54  (0.30 - 0.71) | 0.28  (0.16 - 0.38) |
| eGFR 30-44  ml/min/1.73m^2^ (G3b) | 0.31  (0.23 - 0.38) | 0.22  (0.16 - 0.26) | 0.15  (0.11 - 0.18) | 0.09  (0.06 - 0.10) | 1.25  (0.64 - 1.74) | 0.81  (0.43 - 1.09) | 0.51  (0.28 - 0.68) | 0.26  (0.14 - 0.35) |
| eGFR 15-29  ml/min/1.73m^2^ (G4) | 0.34  (0.25 - 0.43) | 0.23  (0.17 - 0.28) | 0.15  (0.11 - 0.19) | 0.08  (0.06 - 0.10) | 1.22  (0.69 - 1.65) | 0.78  (0.44 - 1.04) | 0.49  (0.27 - 0.65) | 0.23  (0.13 - 0.31) |
| eGFR <15  ml/min/1.73m^2^ (G5) not on RRT | 0.27  (0.19 - 0.33) | 0.21  (0.15 - 0.25) | 0.13  (0.09 - 0.15) | 0.06  (0.04 - 0.07) | 1.16  (0.59 - 1.64) | 0.72  (0.40 - 0.99) | 0.45  (0.25 - 0.61) | 0.19  (0.11 - 0.26) |
| **(b) Predicted additional life years gained with OPTIMAL guideline-indicated use of statins, antihypertensives and antiplatelets in 2019**  **Years (95% confidence intervals)** | | | | | | | | |
| eGFR 60-89 mL/min/1.73^2^ (G2) | 0.32  (0.23 - 0.41) | 0.33  (0.25 - 0.40) | 0.27  (0.21 - 0.33) | 0.18  (0.14 - 0.22) | 0.44  (0.24 - 0.59) | 0.37  (0.20 - 0.50) | 0.26  (0.14 - 0.36) | 0.15  (0.08 - 0.21) |
| eGFR 45-59  ml/min/1.73m^2^ (G3a) | 0.37  (0.25 - 0.47) | 0.34  (0.24 - 0.43) | 0.27  (0.19 - 0.34) | 0.18  (0.12 - 0.22) | 0.50  (0.25 - 0.70) | 0.38  (0.19 - 0.53) | 0.26  (0.13 - 0.37) | 0.14  (0.07 - 0.20) |
| eGFR 30-44  ml/min/1.73m^2^ (G3b) | 0.47  (0.31 - 0.62) | 0.38  (0.25 - 0.49) | 0.28  (0.19 - 0.36) | 0.17  (0.11 - 0.21) | 0.61  (0.29 - 0.87) | 0.41  (0.20 - 0.58) | 0.26  (0.13 - 0.37) | 0.13  (0.06 - 0.18) |
| eGFR 15-29  ml/min/1.73m^2^ (G4) | 0.55  (0.36 - 0.71) | 0.43  (0.29 - 0.54) | 0.30  (0.20 - 0.37) | 0.16  (0.11 - 0.20) | 0.60  (0.31 - 0.83) | 0.41  (0.21 - 0.57) | 0.25  (0.12 - 0.35) | 0.11  (0.05 - 0.16) |
| eGFR <15  ml/min/1.73m^2^ (G5), not on RRT | 0.41  (0.24 - 0.57) | 0.37  (0.25 - 0.48) | 0.25  (0.16 - 0.31) | 0.12  (0.08 - 0.15) | 0.61  (0.28 - 0.89) | 0.39  (0.20 - 0.55) | 0.24  (0.12 - 0.34) | 0.10  (0.05 - 0.14) |
| **Predicted additional quality-adjusted life years gained with OPTIMAL guideline-indicated use of statins, antihypertensives and antiplatelets in 2019**  **QALYs (95% confidence intervals)** | | | | | | | | |
| eGFR 60-89 mL/min/1.73^2^ (G2) | 0.42  (0.34 - 0.48) | 0.37  (0.31 - 0.42) | 0.27  (0.23 - 0.31) | 0.17  (0.14 - 0.19) | 0.46  (0.25 - 0.62) | 0.33  (0.19 - 0.43) | 0.22  (0.13 - 0.29) | 0.12  (0.07 - 0.16) |
| eGFR 45-59  ml/min/1.73m^2^ (G3a) | 0.47  (0.38 - 0.56) | 0.37  (0.29 - 0.44) | 0.28  (0.22 - 0.33) | 0.16  (0.13 - 0.20) | 0.49  (0.27 - 0.65) | 0.34  (0.19 - 0.44) | 0.22  (0.12 - 0.29) | 0.12  (0.07 - 0.16) |
| eGFR 30-44  ml/min/1.73m^2^ (G3b) | 0.53  (0.39 - 0.65) | 0.37  (0.28 - 0.45) | 0.26  (0.19 - 0.31) | 0.14  (0.11 - 0.18) | 0.51  (0.26 - 0.71) | 0.33  (0.18 - 0.45) | 0.21  (0.11 - 0.28) | 0.10  (0.06 - 0.14) |
| eGFR 15-29  ml/min/1.73m^2^ (G4) | 0.59  (0.43 - 0.73) | 0.39  (0.29 - 0.47) | 0.26  (0.20 - 0.32) | 0.13  (0.10 - 0.16) | 0.50  (0.28 - 0.67) | 0.32  (0.18 - 0.42) | 0.20  (0.11 - 0.26) | 0.10  (0.05 - 0.13) |
| eGFR <15  ml/min/1.73m^2^ (G5) not on RRT | 0.45  (0.32 - 0.56) | 0.35  (0.26 - 0.43) | 0.22  (0.16 - 0.26) | 0.10  (0.07 - 0.12) | 0.47  (0.24 - 0.67) | 0.30  (0.16 - 0.40) | 0.18  (0.10 - 0.25) | 0.08  (0.04 - 0.11) |

CVD, cardiovascular disease; eGFR, estimated glomerular filtration rate; NICE, National Institute for Health and Care Excellence; QALY, quality-adjusted life-year; RRT, renal replacement therapy

The predictions are based on a random sample of 64,000 patients from the whole cohort. Of the patients considered to have an indication for cardiovascular prevention treatments in 2019, 37% of those without cardiovascular disease and 71% of those with cardiovascular disease, were treated in 2013. These proportions were calculated using data on patients that were in the study cohort in 2013, and the 2019 UK National Institute for Health and Care Excellence (NICE) guidelines. See Methods and S1 Methods for more information.

## **Methods A.**

**Missing data in the CPRD cohort with reduced kidney function**

Missing data (Table A.1) were either assigned into a separate category (urinary albumin-to- creatinine ratio [uACR]); combined with the base category (ethnicity), or imputed using chained equations (smoking, body mass index [BMI], systolic and diastolic blood pressure, total cholesterol : high density lipoprotein [HDL] cholesterol ratio and Townsend socioeconomic deprivation quintile)[1, 2]. Prior to imputation, the distributions of continuous covariates were tested for normality, and covariates that were not normally distributed were log-transformed. All covariates, as well as the outcome indicators, were included in the imputation models. Categorical variables were imputed with multinomial logistic models and continuous variables were imputed with linear regression models. Five datasets were imputed. Subsequently, all risk equations were fitted on each imputed dataset, and the results were combined using Rubin’s rule[3].

For participants not on renal replacement therapy (RRT), their estimated glomerular filtration rate (eGFR) was calculated from creatinine using the Chronic Kidney Disease Epidemiology collaboration (CKD-EPI) equation[4], and the mean eGFR during that year determined the kidney disease stage. For patients with no creatinine measurements recorded in the annual periods during which they were known to be alive, their latest eGFR status was carried until the next available information. For the cardiovascular and nonvascular death risk equations, categories G5 not on RRT, dialysis and renal transplantation were combined into one category.

Table A.1. Missing data in the CPRD cohort with reduced kidney function

| **Patient’s characteristic** | **Number of participants with data missing at cohort entry (%)** | | | | | |
| --- | --- | --- | --- | --- | --- | --- |
|  | **Total** | eGFR 60-89 mL/min/1.73^2^ (G2) | eGFR 45-59  ml/min/1.73m^2^ (G3a) | eGFR 30-44  ml/min/1.73m^2^ (G3b) | eGFR 15-29  ml/min/1.73m^2^ (G4) | eGFR <15  ml/min/1.73m^2^ (G5), not on RRT |
| Ethnicity | 361,063 (29.9%) | 247,475 (29.2%) | 63,922 (31.02%) | 23,233 (34.12%) | 5,643 (37.06%) | 790 (31.27%) |
| Smoking status | 14,963 (1.3%) | 8,592 (1.01%) | 3,768 (1.83%) | 1,929 (2.83%) | 591 (3.88%) | 83 (3.29%) |
| BMI | 99,848 (8.8%) | 65,513 (7.73%) | 20,730 (10.06%) | 10,161 (14.92%) | 3,027 (19.88%) | 417 (16.51%) |
| Index of multiple deprivation | 3,087 (0.3%) | 2,391 (0.28%) | 520 (0.25%) | 142 (0.21%) | 29 (0.19%) | 5 (0.2%) |
| Systolic blood pressure | 6,955 (0.6%) | 5,295 (0.62%) | 981 (0.48%) | 489 (0.72%) | 169 (1.11%) | 21 (0.83%) |
| Diastolic blood pressure | 6,970 (0.6%) | 5,309 (0.63%) | 982 (0.48%) | 489 (0.72%) | 169 (1.11%) | 21 (0.83%) |
| Total cholesterol : HDL cholesterol ratio | 330,445 (29.0%) | 216,708 (25.57%) | 72,848 (35.35%) | 31,134 (45.72%) | 8,178 (53.71%) | 1,577 (62.43%) |
| Urinary albumin-to-creatinine ratio | 638,929 (56.1%) | 483,664 (57.06%) | 109,997 (53.38%) | 35,368 (51.94%) | 8,278 (54.37%) | 1,622 (64.21%) |

BMI, body mass index; eGFR, estimated glomerular filtration rate; HDL, high-density lipoprotein; RRT, renal replacement therapy

**The lifetime CKD-CVD policy model**

The cardiovascular disease (CVD)/nonvascular death and chronic kidney disease (CKD) submodels were combined into the *lifetime* *CKD-CVD policy model,* a first-order Markov model with an annual cycle of transition (Figure 1). The input to the model consists of individual participant’s characteristics at cohort entry. At start of each cycle, participant’s age and contemporaneous CKD stage and most recent CVD event status are updated. The information initially feeds into the CKD submodel, which updates the participant’s CKD stage for the annual period. This, in turn, enters the CVD/nonvascular death submodel, and the participant’s CVD status in the year is updated. The model then enters the next annual cycle, where the updated disease histories and age are used.

The CKD model states are defined by the patient’s latest eGFR category (G2, G3a, G3b, G4, G5 not on RRT, with renal transplant, dialysis).

The CVD/nonvascular death submodel projects occurrence of strokes and myocardial infarctions (MI) separately, as these events could be differently impacted by interventions. The submodel is based on the patient’s latest cardiovascular disease history and is comprised of five mutually exclusive nonfatal states: no cardiovascular event, nonfatal stroke this year, nonfatal stroke in a previous year, nonfatal MI this year (and no stroke previously) and a nonfatal MI in a previous year (and no stroke previously); and two (absorbing) states: nonvascular death and vascular death. During each annual period in the submodel, events are simulated in the following order: nonvascular death, vascular death, nonfatal stroke and nonfatal MI.

Together, the model states comprise all possible combinations of nonfatal eGFR and CVD states as well as the two fatal (absorbing) states: vascular and nonvascular death and transition probabilities between the states derived from the risk equations, as described in the previous sections.

The annual transitional probabilities in the CVD/nonvascular death submodel are calculated as follows:

1. For all survival data risk equations the annual transitional probability in year *t* is calculated as:

$$p(t)=1-\exp[H(365*(t-1))-H(365*t)],$$

where H(t) denotes the cumulative hazard at the end of year *t*, which is calculated depending on the survival model. For the Weibull model (vascular death or stroke; vascular death, stroke of MI and heart failure hospitalisation equations), the cumulative hazard at time t is H(t)= λt^α^. For the Gompertz model (vascular death and nonvascular death equations), H(t) = λ γ^-1^ (exp(γt) – 1). Here, λ is $\beta_{j}$ a vector of regression coefficients, $x_{j}$ is a vector of covariates, α is the ancillary parameter in the Weibull model and γ is the ancillary parameter in the Gompertz model. A hierarchy of events is imposed in the model so that the probabilities of cardiovascular events (or nonvascular death) are extracted as follows:

1. Nonvascular death (NVD): p(NVD) as per the Gompertz model.
2. Vascular death (VD), conditional on not experiencing (1):

p = p(VD)*[1 – p(NVD)]

1. Nonfatal stroke (stroke), conditional on not experiencing (1)-(2):

p = max[0, p(VD or stroke) – p(VD)] * [1– p(NVD)]

1. Nonfatal MI, conditional on non-experiencing (1)-(3):

p = max[0, p(VD, stroke or MI) – max[p(VD or stroke), p(VD)]] * [1– p(NVD)]

1. To simulate the risk of a heart failure hospitalisation, the Weibull equation is applied to the proportion of population without a previous heart failure hospitalisation.

**Use of statin, antihypertensive and antiplatelet treatment in the cohort in 2013**

Data from the whole study cohort (i.e. estimation and validation cohorts combined) were used to identify patients indicated for treatment with statin, antihypertensive or antiplatelet based on United Kingdom (UK) National Institute for Health and Care Excellent NICE guidance in 2019 [5-7] and assess the percentage who receive treatment. Data for the most recent available year, 2013, were used to summarise patients who received at least two prescriptions during the 2013 calendar year, and indication(s) for treatment on 1 January 2013. Read codes (as opposed to eGFR values) were used to determine presence of chronic kidney disease (Table A.2).

Table A.2. Patients with Read code for chronic kidney disease, indicated for statin, antihypertensive or antiplatelet treatment, and percentage prescribed treatment, 2013

| **Cardiovascular prevention treatment** | **Patients without previous cardiovascular disease** | | **Patients with previous cardiovascular disease** | |
| --- | --- | --- | --- | --- |
|  | Indicated for treatment | % prescribed treatment | Indicated for treatment | % prescribed treatment |
| Statin | 233,373 | 29% | 134,150 | 73% |
| Antihypertensive | 110,674 | 44% | 49,951 | 64% |
| Antiplatelet | 0 | N/A | 134,150 | 76% |

N/A, not applicable

QRISK3 was used to calculate 10-year cardiovascular risk. Due to missing data, QRISK3 score could only be calculated for 67% of the cohort patients without previous cardiovascular disease. To determine eligibility for treatment, for each covariate, data from the latest suitable record on or before 01/01/2013 was used. Prescribed treatment was defined as having had at least two prescriptions between 01/01/2013 and 31/12/2013.

Based on the summaries in Table L, we assumed that among patients indicated for cardiovascular prevention treatments, 37% of those without previous cardiovascular disease and 71% of those with previous cardiovascular disease were receiving the treatment. The effect of treatments (relative risks) on cardiovascular event risks in the model were informed from recent meta-analyses of randomised trials (Table A.3).

**Table A.3. Relative risks with cardioprotective treatments**

| **Treatment and dose mg/day** | **Relative risk (95% CI)** | | | | | | |
| --- | --- | --- | --- | --- | --- | --- | --- |
|  | **eGFR 60-89 ml/min/1.73m^2^**  **(G2)** | **eGFR 45-59**  **ml/min/1.73m^2^**  **(G3a)** | **eGFR 30-44**  **ml/min/1.73m^2^**  **(G3b)** | **eGFR 15-29**  **ml/min/1.73m^2^**  **(G4)** | **eGFR <15**  **ml/min/1.73m^2^**  **(G5) not on RRT** | **Dialysis** |  |
|  |  | | | | | | |
| **Atorvastatin 20mg^1,2^** | | | | | | | |
| Vascular death | 0.78  (0.73, 0.85) | 0.88  (0.80, 0.97) | 0.90  (0.79, 1.02) | 0.81  (0.69, 0.95) | 0.82  (0.70, 0.96) | 1.00  (0.87, 1.15) |  |
| Myocardial infarction | 0.65  (0.60, 0.69) | 0.64  (0.57, 0.73) | 0.74  (0.61, 0.91) | 0.82  (0.62, 1.09) | 0.83  (0.63, 1.09) | 0.85  (0.66, 1.10) |  |
| Stroke | 0.77  (0.70, 0.85) | 0.75  (0.65, 0.87) | 0.89  (0.72, 1.10) | 0.80  (0.62, 1.03) | 0.81  (0.63, 1.09) | 1.11  (0.86, 1.42) |  |
| **Atorvastatin 40mg^1,2^** | | | | | | | |
| Vascular death | 0.77  (0.71, 0.83) | 0.87  (0.78, 0.97) | 0.89  (0.78, 1.02) | 0.79  (0.66, 0.95) | 0.80  (0.67, 0.95) | 1.00  (0.86, 1.17) |  |
| Myocardial infarction | 0.62  (0.57, 0.67) | 0.62  (0.54, 0.71) | 0.72  (0.58, 0.90) | 0.81  (0.59, 1.10) | 0.81  (0.60, 1.10) | 0.84  (0.63, 1.11) |  |
| Stroke | 0.75  (0.67, 0.84) | 0.73  (0.62, 0.86) | 0.88  (0.70, 1.10) | 0.78  (0.59, 1.03) | 0.79  (0.60, 1.03) | 1.12  (0.85, 1.47) |  |
| **Atorvastatin 80mg^1,2^** | | | | | | |  |
| Vascular death | 0.75  (0.68, 0.82) | 0.86  (0.76, 0.97) | 0.88  (0.76, 0.97) | 0.77  (0.63, 0.94) | 0.78  (0.65, 0.95) | 1.00  (0.84 1.19) |  |
| Myocardial infarction | 0.59  (0.54, 0.64) | 0.58  (0.50, 0.68) | 0.70  (0.55, 0.89) | 0.79  (0.56, 1.11) | 0.80  (0.57, 1.11) | 0.82  (0.60, 1.13) |  |
| Stroke | 0.73  (0.65, 0.83) | 0.71  (0.59, 0.85) | 0.87  (0.67, 1.12) | 0.76  (0.56, 1.04) | 0.77  (0.57, 1.04) | 1.13  (0.84, 1.53) |  |
| **Angiotensin-converting enzyme (ACE) inhibitors^3,5^** | | | | | | |  |
| Vascular death | 0.87  (0.78, 0.98) | 0.80  (0.69, 0.93) | 0.80  (0.69, 0.93) | 0.80  (0.69, 0.93) | 0.80  (0.69, 0.93) | 0.80  (0.69, 0.93) |  |
| Myocardial infarction | 0.79  (0.75 0.87) | 0.81  (0.71, 0.93) | 0.81  (0.71, 0.93) | 0.81  (0.71, 0.93) | 0.81  (0.71, 0.93) | 0.81  (0.71, 0.93) |  |
| Stroke | 0.85  (0.71, 1.03) | 0.81  (0.68, 0.96) | 0.81  (0.68, 0.96) | 0.81  (0.68, 0.96) | 0.81  (0.68, 0.96) | 0.81  (0.68, 0.96) |  |
| Heart failure hospital admission | 0.90  (0.74, 1.09) | 0.75  (0.63, 0.91) | 0.75  (0.63, 0.91) | 0.75  (0.63, 0.91) | 0.75  (0.63, 0.91) | 0.75  (0.63, 0.91) |  |
| **Aspirin 75mg^4,5^** | | | | | | |  |
| Vascular death | 0.87  (0.65, 1.15) | 0.87  (0.65, 1.15) | 0.87  (0.65, 1.15) | 0.87  (0.65, 1.15) | 0.87  (0.65, 1.15) | 0.87  (0.65, 1.15) |  |
| Myocardial infarction | 0.68  (0.46, 0.99) | 0.68  (0.46, 0.99) | 0.68  (0.46, 0.99) | 0.68  (0.46, 0.99) | 0.68  (0.46, 0.99) | 0.68  (0.46, 0.99) |  |
| Stroke | 1.00  (0.58, 1.72) | 1.00  (0.58, 1.72) | 1.00  (0.58, 1.72) | 1.00  (0.58, 1.72) | 1.00  (0.58, 1.72) | 1.00  (0.58, 1.72) |  |

eGFR, estimated glomerular filtration rate; RRT, renal replacement therapy

^1^Ballantyne CM, Abate N, Yuan Z, King TR, Palmisano J. Dose-comparison study of the combination of ezetimibe and simvastatin (Vytorin) versus atorvastatin in patients with hypercholesterolemia: the Vytorin Versus Atorvastatin (VYVA) study. Am Heart J. 2005; 149(3):363-73 *(Table II: % change from baseline for LDL-C)*

^2^Cholesterol Treatment Trialists’ Collaboration, Impact of renal function on the effects of LDL cholesterol lowering with statin-based treatments: a meta-analysis of individual participant data from 28 randomised trials. Lancet Diabetes Endocrinol. 2016;4(10):829-839

^3^Blood Pressure Lowering Treatment Trialists’ Collaboration. Blood pressure lowering and major cardiovascular events in people with and without chronic kidney disease: meta-analysis of randomised controlled trials. BMJ. (2013):357:f5680.

^4^Palmer SC, Di Micco L, Razavian M, et al. Antiplatelet agents for chronic kidney disease. Cochrane Database Syst Rev 2013; (2): CD008834

^5^Patients on transplant assumed to have the same treatment effects as those in G3a in lieu of other information

The relative risks with statin treatment by eGFR category^2^ were weighted by the absolute reduction in LDL cholesterol, derived using proportional reductions in LDL cholesterol with the particular atorvastatin dose^1^ and LDL cholesterol of patients in the respective eGFR category at cohort entry (Table C).

1. Royston P, White IR. Multiple Imputation by Chained Equations (MICE): Implementation in Stata. 2011. 2011;45(4):20.

2. Royston P. Multiple imputation of missing values: Further update of ice, with an emphasis on categorical variables. Stata Journal StataCorp LP. 2009;9(3):466--77.

3. Rubin DB. Multiple Imputation for Nonresponse in Surveys: Wiley; 2004.

4. Levey AS, Stevens LA, Schmid CH, Zhang YL, Castro AF, 3rd, Feldman HI, et al. A new equation to estimate glomerular filtration rate. Ann Intern Med. 2009;150(9):604-12.

5. National Clinical Guideline Centre (UK). Lipid Modification: Cardiovascular Risk Assessment and the Modification of Blood Lipids for the Primary and Secondary Prevention of Cardiovascular Disease. NICE Clinical Guidelines, No. 181. London: National Institute for Health and Care Excellence (UK); 2014.

6. National Clinical Guideline Centre (UK). Chronic kidney disease in adults: assessment and management. NICE Clinical Guidelines, No. 182. London: National Institute for Health and Care Excellence (UK); 2014.

7. National Clinical Guideline Centre (UK). Hypertension in adults: diagnosis and management. NICE guideline [NG136]. London: National Institute for Health and Care Excellence (UK); 2019.
