# Supplementary material for: Long-term health outcomes of people with reduced kidney function in the UK: A modelling study using population health data
Source: PLoS Med. 2020 Dec 16;17(12):e1003478. doi: 10.1371/journal.pmed.1003478 (PMC7769604; doi:10.1371/journal.pmed.1003478)
Supplement: Attachment — Submitted filename: Response to reviewers.docx [file pmed.1003478.s003.docx]

**Authors’ response to editors and reviewers comments**

**Editors’ comments**

1. **Please revise your title according to PLOS Medicine's style. Your title must be nondeclarative and not a question. It should begin with main concept if possible. "Effect of" should be used only if causality can be inferred, i.e., for an RCT. Please place the study design ("A randomized controlled trial," "A retrospective study," "A modelling study," etc.) in the subtitle (ie, after a colon).**

Done. We have revised the manuscript title to “Long-term health outcomes of people with reduced kidney function: a policy model derived from a large UK population cohort”

2. **Abstract**

**Abstract Background: Provide the context of why the study is important. The final sentence should clearly state the study question.**

**Please include the study design, population and setting, number of participants, years during which the study took place, length of follow up, and main outcome measures.**

**Please quantify the main results (with 95% CIs and p values).**

**Please include the important dependent variables that are adjusted for in the analyses.**

**In the last sentence of the Abstract Methods and Findings section, please describe the main limitation(s) of the study's methodology.**

**Abstract Conclusions:**

*** Please address the study implications without overreaching what can be concluded from the data; the phrase "In this study, we observed ..." may be useful.**

*** Please interpret the study based on the results presented in the abstract, emphasizing what is new without overstating your conclusions.**

*** Please avoid vague statements such as "these results have major implications for policy/clinical care".**

**Mention only specific implications substantiated by the results.**

*** Please avoid assertions of primacy ("We report for the first time....")**

Done. The abstract has been revised as per editors’ guidance - clean and tracked edited versions submitted.

3. **Author summary**

**At this stage, we ask that you include a short, non-technical Author Summary of your research to make findings accessible to a wide audience that includes both scientists and non-scientists. The Author Summary should immediately follow the Abstract in your revised manuscript. This text is subject to editorial change and should be distinct from the scientific abstract. Please see our author guidelines for more information:** [**https://journals.plos.org/plosmedicine/s/revising-your-manuscript#loc-author-summary**](https://journals.plos.org/plosmedicine/s/revising-your-manuscript#loc-author-summary)

Done. We have included author summary with revised paper in the manuscript (after Abstract).

4. **References- please use square brackets and update the bibliography to Vancouver style. Please place the full stop after the square brackets.**

Done.

**Lines 188-191 could you provide the sources of these external data please? It would be clearer to mention the names of data sets and provide citations.**

References are included in the S1 Table and S2 Table in S1 Supporting Information. We have edited manuscript text to make this clearer, see page 12 lines 7-10.

5. **Please can you ensure all acronyms are introduced on first view**

Done

6. **Discussion**

**Please present and organize the Discussion as follows: a short, clear summary of the article's findings; what the study adds to existing research and where and why the results may differ from previous research; strengths and limitations of the study; implications and next steps for research, clinical practice, and/or public policy; one-paragraph conclusion.**

**Please include a brief summary of clinical implications of the model and your findings, as this section focusses heavily on the model development/validation aspects.**

Done. Specifically, we have added paragraphs about study’s strengths, key areas of further development and have substantially develop the final paragraph to summarise clinical implications of the model and findings (see pages 18-20).

7. **Please ensure that the study is reported according to the TRIPOD guideline, and include the completed checklist as Supporting Information. When completing the checklist, please use section and paragraph numbers, rather than page numbers. Please add the following statement, or similar, to the Methods: "This study is reported as per the XXX guideline (S1 Checklist)."**

Done, see page 7 lines 7-9 and S1 checklist.

8. **Did your study have a prospective protocol or analysis plan? Please state this (either way) early in the Methods section.**

**a) If a prospective analysis plan (from your funding proposal, IRB or other ethics committee submission, study protocol, or other planning document written before analyzing the data) was used in designing the study, please include the relevant prospectively written document with your revised manuscript as a Supporting Information file to be published alongside your study, and cite it in the Methods section. A legend for this file should be included at the end of your manuscript.**

**b) If no such document exists, please make sure that the Methods section transparently describes when analyses were planned, and when/why any data-driven changes to analyses took place.**

**c) In either case, changes in the analysis-- including those made in response to peer review comments-- should be identified as such in the Methods section of the paper, with rationale**.

We prospectively developed a protocol for the wider programme of work, covering the CPRD-based work in our NIHR Programme Grant (see Funding Section). The protocol (enclosed with this revision) was reviewed and approved by the Independent Scientific Advisory Committee (ISAC) of the Medicines and Healthcare Products Regulatory Agency. Whilst this protocol covered some aspects of the study, reported in the manuscript, including description of the study population and definition of kidney disease progression, the level of information required that is relevant to this manuscript was very limited. Therefore, it is our view that it would not be relevant as an appendix for this manuscript. We have however amended the text to indicate that the protocol is available on request, see page 7 line 11.

**Reviewer 1 comments**

1. **The authors describe a policy model they have developed for evaluating the long-term effects of cardiovascular interventions in patients with varying levels of reduced kidney function. This is very interesting work, which addresses highly clinically relevant questions.**

Thank you.

2. **I was not able to fully follow how the models were developed based on the primary text and the supporting information. (I was not able to figure out how to access the Supplementary Information, which sounds like it might be been a separate document from the Supporting Information, however.) In part, this is likely because the modelling is complex, and it may not be possible to provide a complete description of the models and their assumptions within the scope of an article such as this. However, it is possible that some of the main concepts and assumptions underlying the models could be articulated more clearly, and that the authors may be able to better describe the impact of underlying assumptions of the models on their results.**

We apologise for the confusion. We have now clearly signposted the Supporting Information and relevant separate sections therein throughout the manuscript, see page 9 line 6.

**With this perspective, I'll list a few of the questions that occurred to me as I read the article in case they are of use to the authors:**

**1) It appeared from the description of the CKD submodel and the Integrated CKD-CVD model that the authors used the data from their cohort to estimate 1-year transition probabilities between the different CKD and CVD states as a function of various baseline covariates, and then used Markovian assumptions to model the state probabilities over time. However, the description of the CVD/non CV death submodel appears to be a Anderson-Gill survival model, was gives the impression of a model that relates the overall time-to-events to baseline covariates, as opposed to estimation of 1-year transition probabilities. This apparent contradiction in approaches left me confused. I think it would be useful to indicate if in fact all modeling for both the CKD and the CVD states was based on development of 1-year transition probabilities between states, where the transition rates were allowed to depend on the covariates that were indicated in the manuscript. And if so, how did the**

**Anderson-Gill analyses for the CVD/non CV death model inform this transition rates?**

We have further clarified this in the manuscript. Transition probabilities were derived from the individual risk equations (Anderson-Gill generalisation of Cox survival models for the CV submodel). We have added this information in the Methods, see page 11 lines 17-19 and S1 Methods / The lifetime CKD-CVD policy model, paragraph 4.

**2) How were the different types of events accounted for in the Anderson Gill models that evaluated multiple event times?**

The Anderson-Gill generalisation of the Cox model enables inclusion of time updated characteristics (patient’s age and cardiovascular and CKD endpoints in our model). First event of each type was modelled, and events from other types that occurred earlier contributed to the estimations (e.g. an MI that happened before a vascular death would be included in the vascular death equation). We have added further detail in the Methods, see page 9 line 21 – page 10 line 6.

**3) If in fact the modeling was done based on 1-year transition probabilities between CVD and CKD states, I would have thought that it would also be necessary to model longitudinal changes from year to year in the various patient characteristics that inform the transition probabilities. Alternatively, if the transition rates are model in terms of the baseline patient characteristics only, I would think it would be necessary to allow the transition rates (for given values of the baseline characteristics) to change over time. For example, sure the risk of transitioning to a worse state at a given baseline proteinuria would differ for the 1st year and the 20th year after baseline. But I didn't see any of this mentioned in the text. I think it would be useful to clarify.**

Whilst many patient characteristics were fixed at baseline, the key risk factors, i.e. age, cardiovascular events and eGFR category, were updated annually using the longitudinal data for the purpose of estimating the risk equations, and are predicted annually in the model. The baseline characteristics contribute to risks of events (e.g. incidence of cardiovascular event), which, in turn, inform risks of further events and mortality. We have added further detail in the Methods (see page 10 lines 3-6) and also moved some information from S1 Methods (S1 Supporting Information) into the main text, see page 11 line 24 - page 12 line 3 (also S1 Methods / The lifetime CKD-CVD policy model, paragraph 1).

**4) It appears the Markov models were based on transition probabilities assuming none of the CVD interventions were in place for the untreated condition, and then the authors incorporate estimates of the treatment effects for these interventions based on the literature to estimate transition probabilities for the treated patients. Since many of the patients in the actual cohort were treated, how were transition probabilities for untreated patients estimated?**

Yes, many patients in the cohort were treated with cardioprotective medications. This information was extracted at entry, and treatment indicators were included as covariates in the risk equations (separate indicators for the use of statins, antiplatelets and antihypertensives). This information was included in the footnotes to the Tables; it is now also included in the Methods section, see page 10 lines 6-7. In the model, effects of treatments were simulated with effects informed from randomised trials, see our response to comment 5.

**5) When incorporating treatment effects from the literature, were treatment effects assumed constant for all patients on a hazard ratio or relative risk scale, as is commonly assumed? Or were they assumed constant on an absolute risk scale? Or was some other assumption used? Were any treatment by covariate interactions assumed, or were the treatment effects assumed constant (on one of the scales noted above) across different patient characteristics? Were the treatment effects assumed constant over time, or was some attenuation in the effect of the treatment over time assumed? These all seem like very fundamental assumptions that are likely to strongly affect the results that were presented, and I think it may be useful to summarize the assumptions that were made, and their likely implications for the results that were presented.**

Thank you for flagging the previously limited detail included. The relative treatment effects were assumed constant over time and taken from meta-analyses of randomised clinical trials. We have added further details and a new Supporting Table (Table SM3) with this information in S1 Methods in S1 Supporting Information, as well as noted this in the manuscript (see page 14 lines 1-3).

**Reviewer 2 comments**

1. **Thanks for the opportunity to review your manuscript. My role is as a statistical reviewer so my queries and comments are concentrated on the analysis and data, and the reporting of these. This is a very interesting manuscript, which selects of a cohort of people with impaired kidney function and then uses the outcomes of this cohort to predict CKD and CV and uses these risk prediction models in a Markov model to look at implications different treatment options. The manuscript is clearly written and with useful supplementary information about the models underlying the key results reported. I have some overall queries about the analysis, followed by ones specific to a section of the manuscript.**

Thank you.

2. **An overall query I had was the whether the intention of the manuscript methodological (development and validation of the tool) or is it concentrated on the results of the Markov model? I appreciate that this is a complex analysis and with limited space it's difficult to give a detailed context for kidney impairment and the current state of treatment options for patients. There is probably a series of papers that could come from this work (a 'treatment gap' analysis would be very interesting).**

Thank you for your comment. The primary focus of the paper is methodological, presenting the model. The two substantive data-driven applications, including the ‘treatment gap’ analysis, illustrate the model use and its potential to inform policy. We have revised the Abstract and the Discussion section to clarify this.

3. **I may have missed it but I couldn't find a table describing the patterns of missingness for the covariates used in the study by eGFR status - Table SM1 is helpful and if possible to get this by the kidney function patients this gives a sense of limitations of the CPRD data. The approach to missing data is explained well, and the limitations of the possibility of data being MNAR is also acknowledged in the discussion. I don't think there is an obvious MNAR sensitivity analysis that could be implemented with this data and analysis.**

We have now extended Table SM1 in S1 Methods (S1 Supporting Information) and included the patterns of missingness by eGFR status.

4. **Only 5 imputations were used, this is a relatively low number. What criteria was used to decide the number of imputations, i.e. a quantitative measure like fraction of missing information or was the small number of imputations necessary because of the size of the dataset (my own personal experience is that both Stata and R are very capable with MI but the process is extremely time-consuming with a large dataset).**

The number of imputations was chosen as a compromise between accuracy and computation time. Whilst both R and Stata are indeed capable of performing the MI procedure, it was very time-consuming to perform it on a 1.1-million dataset (~15 hours).

5. **Are the risk prediction models available for use by other researchers, e.g. as PMML or as R-code?**

The risk equations are fully reported in manuscript. We have now made available the R code of the CKD-CVD Markov model, which includes these equations, in a public repository <https://github.com/ischlackow/CKD-CVD-routine-data-model> , and added reference to in the manuscript, see page 20 lines 1-4.

6. **Similarly is the code for the other models (CKD submodel and Markov model) available?**

The code for the CKD submodel is available as part of the CKD submodel paper; this is now noted in manuscript page 20 lines 3-4. The code of the CKD-CVD Markov model, including use of the CKD submodel, has now been placed into a public repository; see our response to comment 5.

7. **The c-index shows that the predictive models perform better in patients with better kidney function. What are the consequences for the Markov models when the discrimination of the risk models is lower?**

This, together with model validation results, indicates that, whilst the model performs well at eGFR category level, its performance at individual level among participants with more advanced kidney function impairment is less reliable/does not differentiate risk as well among patients in these categories. We have now made this point in the discussion of limitations, see page 19, lines 8-9.

8. **P5, L100. Is the 'up to standard' a general quality measure for CPRD data or is this specific to this study?**

This is the general minimum data quality standard at general practice level in CPRD, i.e. meeting the CPRD standard for continuity of recording and number of recorded deaths. This is now clarified on page 8 lines 3-4.

9. **P5, L106. Is natural decline in kidney function with age incorporated into the selection criteria or is this likely to have a minimal impact on finding cases?**

No. Natural decline in kidney function with age does not affect CKD management recommendations and, therefore, no age-specific inclusion was applied.

10. **In Supp Fig 1 there are ~75k patients excluded as they had abnormal tests less than 90 days apart - is this used as an indicator of AKI or suspected AKI? When results are this close together are the patients then excluded completely (even if they have a long-term pattern of kidney impairment) or are these tests just not considered to be evidence of CKD? My understanding is that long-term loss of function is considered to be a potential outcome of AKI even after recovery in the short-term.**

First occurrence of any (not necessarily consecutive) two eGFR tests <90 mL/min/1.73 m2 at least 90 days apart qualify individual for inclusion. This assures that long-term pattern of kidney impairment is captured. Two measures at least 90 days apart is based on the definition of CKD used both in KDIGO and NICE definitions of CKD, which require a kidney damage and/or reduced eGFR to be present for at least 3 months. We have added the references in the Methods (see References 14 and 17). AKI itself does not trigger specific cardioprotective or other therapy in primary care and whilst it may herald CKD, is not in itself relevant to the current study. If AKI led to CKD, then this would be picked up by the algorithm applied.

11. **P5, L115. Could a patient be censored when they transfer out of one practice, and then enter the cohort (as a 'different' patient) again if they meet the inclusion criteria at a new practice?**

This may indeed happen and we have no way of identifying it. However, we expect this to be a (relatively) rare event given that CPRD data in the study are based on a small proportion of all GP practices in the country (~8%) and movement of a patient from one CPRD practice to another GP practice also contributing into CPRD is relatively unlikely.

12. **P5. L135. Does this refer to missing uACR being accounted for using the missing-as-indicator approach?**

Yes. See also our reply to Reviewer 3, Comment 5.

13. **P5, L152. What specific algorithm using AIC was used?**

Apologies, this was a typo and AIC was used only in selection of parametric survival distribution, as noted later in paragraph (page 10 line 23).Variable selection was performed with the Stata *stepwise* command, specifying both backward (ie, pr (0.05)) and forward (ie, pe(0.01)) parameters. We have now removed the mention of AIC from the covariate selection sentence (page 10 lines 13-14).

14. **P5. L153. Was an alpha of 0.01 used as a cut-point to determine statistical significance? This is mentioned a few lines later but I couldn't tell if this was the criteria used in this step.**

Yes. We have added this in the text, see page 10 line 15.

**What was the criteria for 'clinical' significance'?**

Clinical advice from collaborators during review of risk equations. Typically risk factors with more marginal significance (p-values 0.01-0.05) were considered at this stage.

15. **P5. L157. So model selection was done with the recurrent-events analysis, and then a parametric survival model was estimated including the variables identified from the initial model selection?**

Yes, model selection was performed with time updated covariates included. Note that for each type of event, only the first event was included/ modelled. The parametric survival model was selected subsequently.

16. **P10, L213. Typo in 'szmpled' - should be sampled?**

Thank you, this has been corrected.

**Reviewer 3 comments**

1. **Overall, this is an excellent paper that has done a really nice job in summarising a complex policy model and its development. A commendable transparent reporting approach has been taken and the others are making their codes available. However, I think there remain some important issues to address in terms of justifying the methods used and in the reporting.**

Thank you. Please note that we have now made the source code available from <https://github.com/ischlackow/CKD-CVD-routine-data-model>; see also our reply to Review 2, Comments 5 and 6.

2. **Introduction: Excellent; appropriate length; sets the scene well and the rationale for the research is clear.**

Thank you.

3. **Methods:Cohort definition: what was rationale of patient having to have at least two eGFR tests at least 90 days apart?**

This is based on the KDIGO guidelines. See also our reply to Review 2, comment 10.

4. **Medication / intervention: from CPRD you can access pharmacological and other intervention information. I don’t think individual’s intervention use (CVD or otherwise) was accounted for in the risk prediction models? This would seem like an important consideration as likely to influence outcomes?**

Thank you. Yes, this is an important consideration and was accounted for in risk models. All cardiovascular risk equations included adjustments for use of key cardioprotective medications at baseline. See also our response to Review 1, Comment 4.

5. **Missing data: why was uACR treated differently to the other covariates? I’m guessing because has over 50% missing data but there is evidence that MI can ‘handle’ variables with large amounts of missing data (e.g. Janssen et al; Journal Clinical Epi 63 (2010): 721-727).**

Yes, the degree of missing data was high and uACR was not missing at random. We felt therefore that multiple imputation methods may not be appropriate, especially since we are interested in predictive modelling. We have now discuss this point in the text, see page 18 lines 19-20. Also, the CKD model was estimated (and simulated) separately among patients with missing uACR, see page 11 lines 4-5.

6. **Deriving risk equations: excellent that data held back for internal validation purposes. Why was this done in a random way? Why would you expect performance to be different in a randomly chosen subset? Did you consider doing this in a non-random way?**

Thank you for this comment. This was done to preserve direct generalisability of model to the whole population. In addition, model performance was also checked separately in the ten geographical regions, see results presented in S3 Table in S1 Supporting Information.

7. **Uncertainty: parameter uncertainty is addressed but what about structural uncertainty? How were the Markov model states determined and for what reasons? Why was a cohort modelling approach taken instead of an individual-based simulation approach (e.g. DES)?**

Model states were determined in view of the key aim of the model to address interventions to reduce CVD risk and CKD progression. Whilst we did consider DES, we decided to implement a less computationally intensive approach instead to focus on patient heterogeneity (run results for categories of actual patients’ profiles) and facilitate estimation of uncertainty. We recognise however that DES enables easier incorporation of further endpoints and have suggested this as a possibility for further research, see page 19 lines 15-18.

8. **LE validation: I think it is important to compare the remaining LEs with national lifetables. From Supp Table 9, some of the predictions look on the high-side (e.g. eGFR 60-89, <60 years old with CVD expected to live until 92 years?).**

We have done this, but the comparison is too rough to directly include in the manuscript (i.e., we failed to identify suitable lifetable data in people with and without CVD separately). Please note that in this youngest age category, the mean age is 49 years, for which LE in the general population is 84 (male)/87 (female) years according to <https://www.ons.gov.uk/peoplepopulationandcommunity/healthandsocialcare/healthandlifeexpectancies/articles/lifeexpectancycalculator/2019-06-07>. Comparisons in other age categories are more distanced and this is expected with. As such, we believe that results are generally as expected, i.e. life expectancy estimates of the cohort population are lower than that of general population across age categories. See also page 17 lines 20-23 in the Discussion comparing our life expectancy estimates with those obtained with a previous model, which was based on moderate-to-severe CKD.

9. **Minor: number of cause of death fields is detailed – do the same for diagnosis fields in HES?**

If we understand the comment correctly, it is suggested to use all ICD10 codes (i.e. both primary and secondary) at discharge to indicate presence of cardiovascular disease, which is exactly what was done. We have now made this clearer in the text, see page 7 line 18-19.

10. **Minor: CPRD data – “good quality data” is vague; can a little more detail be provided on how this is defined / add a reference?**

Done. See our response to Reviewer 2 comment 8.

11. **Results: I think it is important to report what percentage of the total study cohort did not have any transition during the observed follow-up period (i.e. leaving the starting state) in the opening paragraph. This shows how ‘hard’ the extrapolations have to work.**

The number of cardiovascular endpoints by eGFR stage are presented in S5 Table in S1 Supporting Information. The degree of transitions from each eGFR stage to another have been published in the CKD submodel paper (see Reference 20). We have noted this in the text, see page 15 lines 1-2.

12. **Discussion: Very well measured. The limitations are clearly laid out and the implications of them discussed in a balanced way. I think there should be a section on ‘strengths’ as it is warranted!**

Thank you, we have now added a section on strengths, see page 18 lines 5-13.

**Other requests**

**In revising the manuscript for further consideration, your revisions should address the specific points made by each reviewer and the editors. Please also check the guidelines for revised papers at**

**http://journals.plos.org/plosmedicine/s/revising-your-manuscript for any that apply to your paper.**

**In your rebuttal letter you should indicate your response to the reviewers' and editors' comments, the changes you have made in the manuscript, and include either an excerpt of the revised text or the location (eg: page and line number) where each change can be found. Please submit a clean version of the paper as the main article file; a version with changes marked should be uploaded as a marked up manuscript.**

Done.

**In addition, we request that you upload any figures associated with your paper as individual TIF or EPS files with 300dpi resolution at resubmission; please read our figure guidelines for more information on our requirements: http://journals.plos.org/plosmedicine/s/figures. While revising your submission, please upload your figure files to the PACE digital diagnostic tool, https://pacev2.apexcovantage.com/.**

**PACE helps ensure that figures meet PLOS requirements. To use PACE, you must first register as a user.**

**Then, login and navigate to the UPLOAD tab, where you will find detailed instructions on how to use the tool. If you encounter any issues or have any questions when using PACE, please email us at**

[**PLOSMedicine@plos.org**](mailto:PLOSMedicine@plos.org)**.**

Done.

**We expect to receive your revised manuscript by Jul 30 2020 11:59PM. Please email us**

**(plosmedicine@plos.org) if you have any questions or concerns.**

*****Please note while forming your response, if your article is accepted, you may have the opportunity to make the peer review history publicly available. The record will include editor decision letters (with reviews) and your responses to reviewer comments. If eligible, we will contact you to opt in or out.*****

Noted.

**We ask every co-author listed on the manuscript to fill in a contributing author statement, making sure to declare all competing interests. If any of the co-authors have not filled in the statement, we will remind them to do so when the paper is revised. If all statements are not completed in a timely fashion this could hold up the re-review process. If new competing interests are declared later in the revision process, this may also hold up the submission. Should there be a problem getting one of your co-authors to fill in a statement we will be in contact. YOU MUST NOT ADD OR REMOVE AUTHORS UNLESS YOU HAVE ALERTED THE EDITOR HANDLING THE MANUSCRIPT TO THE CHANGE AND THEY SPECIFICALLY HAVE AGREED TO IT. You can see our competing interests policy here:**

**http://journals.plos.org/plosmedicine/s/competing-interests.**

Noted. There are no changes to authorship.

**To enhance the reproducibility of your results, we recommend that you deposit your laboratory protocols in protocols.io, where a protocol can be assigned its own identifier (DOI) such that it can be cited independently in the future. For instructions see**

[**http://journals.plos.org/plosmedicine/s/submission-guidelines#loc-methods**](http://journals.plos.org/plosmedicine/s/submission-guidelines#loc-methods)**.**

N/A

**Please ensure that the paper adheres to the PLOS Data Availability Policy (see**

**http://journals.plos.org/plosmedicine/s/data-availability), which requires that all data underlying the study's findings be provided in a repository or as Supporting Information. For data residing with a third party, authors are required to provide instructions with contact information for obtaining the data. PLOS journals do not allow statements supported by "data not shown" or "unpublished results." For such statements, authors must provide supporting data or cite public sources that include it.**

Done. We have added sections on Software availability (page 20 lines 1-4) and data availability (page 20 lines 5-11)
