# Supplementary material for: Long-term health outcomes of people with reduced kidney function in the UK: A modelling study using population health data
Source: PLoS Med. 2020 Dec 16;17(12):e1003478. doi: 10.1371/journal.pmed.1003478 (PMC7769604; doi:10.1371/journal.pmed.1003478)
Supplement: Attachment — Submitted filename: Response to editors comments.docx [file pmed.1003478.s004.docx]

**Authors’ response to editors and reviewers comments**

**Editors’ requests**

1. **Title : Please revise to "Long-term health outcomes of people with reduced kidney function in the UK: a modelling study" to include a study descriptor in accordance with PLOS Medicine style**

Done. We have revised the manuscript title to “Long-term health outcomes of people with reduced kidney function in the UK: a modelling study using population health data”. Unlike most modelling studies which are based on published summary data, the model presented is fully informed from a large population health data and this needs to be reflected in manuscript title as an important study design feature.

2. **Abstract-Please provide an age range for participants included in your cohort**

Done. We have edited to “cohort of adults (≥18 years of age)”.

3. **Abstract- limitations should be explicitly noted at the end of the methods and findings, for example “limitations include xxx”. Please also provide 2-3 limitations**

Done. We have added “Limitations include risk factor measurements from UK routine primary care database and limited albuminuria measurements.”.

4. **Abstract- could you define “optimal cardioprotective medication use”?**

Done. We edited the text to “optimal (i.e. fully compliant with clinical guidelines in 2019) use of cardioprotective medications”.

5. **"Substantial benefit" is perhaps a bit of an overstatement that should be toned down in the abstract and elsewhere towards the end of the abstract, suggest that substituting actual numbers for "more than doubled" and "increased by a third" would be better.**

Done. We have edited to “increased by further 0.33 (0.25-0.40) and 0.37 (0.20-0.50) years, respectively” and “*clear* survival benefits with cardioprotective treatments in this population”

6. **Author summary: Line 5-6 could you revise to specify for whom this is a key target?**

Done. We have clarified that this refers to “people with chronic kidney disease”.

7. **Author summary: Line 15-16 needs to be revised for clarity, please specify what categories were used and what model performance means?**

Done. We have edited this point to “The model achieved good risk discrimination and accurately predicted risks of cardiovascular events in patient categories by kidney function impairment (estimated glomerular filtration rate [eGFR] 60-89; 45-59; 30-44; 15-29; and <15 ml/min/1.73m^2^ not on renal replacement therapy) and by ten geographic regions in England”.

8. **Page 9 line 12- I suggest this is either removed or moved to the data availability statement. PLOS data policy requires that the data underlying the findings be provided in a repository or as supplementary information unless there are ethical restrictions to data-sharing. If there are restrictions on sharing codelists and algorithms, please provide these in the data availability statement also noting the contact details for who may be able to provide these to interested parties. Please note the sole contact cannot be one of the study authors.**

Done. The codelists/algorithms will be made available together with model R code at <http://www.herc.ox.ac.uk/downloads/supportingmaterial> at manuscript publication. This is noted at end of manuscript in section “Software and codelists availability”.

9. **Page 20 line 4-6 please rephrase “likely to be used by” to “may be beneficial to” to avoid overstating utility**

Done. The phrase was edited to “The model may be beneficial to…”.

10. **Please add an additional sentence to the paragraph beginning the discussion to summarize the study's Findings**

Done. We have added “In the current study, we present a novel policy model of kidney disease progression and cardiovascular complications in people with reduced kidney function, and quantify the benefits of cardioprotective medications in this population. The model demonstrates good disease risk discrimination and predictive accuracy and can be used to…”.

11. **Funding information/competing interests and data availability statements can be removed from the main text as these are provided in the article meta-data**

Done.

12. **Please provide access details for ref 32**

Reference detail is now completed.

**Comments from reviewers**

The reviewers were satisfied with our responses and did not raise further queries.
